# Development of a counterselection system for efficient marker-free genetic manipulation in *Avibacterium paragallinarum*

**DOI:** 10.1128/aem.02398-25

**Published:** 2026-03-30

**Authors:** Juan Sun, Ling Chen, Ge He, Yizhen Lin, Jialian Hu, Ning Dai, Xuewei Cao, Yifeng Huang, Yu Han, Saixiang Feng

**Affiliations:** 1 College of Veterinary Medicine, South China Agricultural University 554665, Guangzhou, China; 2 Key Laboratory of Zoonosis Prevention and Control of Guangdong Province 12526, Guangzhou, China; 3 Key Laboratory of Zoonosis of Ministry of Agriculture and Rural Affairs, Guangzhou, China; 4 Key Laboratory of Veterinary Vaccine Innovation of the Ministry of Agriculture and Rural Affairs, Guangzhou, China; 5 National and Regional Joint Engineering Laboratory for Medicament of Zoonosis Prevention and Control, Guangzhou, China; Washington University in St Louis, St. Louis, Missouri, USA

**Keywords:** *Avibacterium paragallinarum*, *Pasteurellaceae*, marker-free mutagenesis, counterselectable marker, *pheS*

## Abstract

*Avibacterium paragallinarum* causes infectious coryza in chickens, a condition that is prevalent worldwide and causes considerable losses for the poultry sector. However, the scarcity of information concerning the virulence factors of the bacterium and the underlying pathogenic mechanisms is largely due to the lack of effective and practical tools for genetic manipulation. Herein, the point mutation A294G was introduced in the gene *pheS* of *Av. paragallinarum* to establish a counterselectable marker *AppheS*m. The host strain harboring the gene *AppheS*m exhibited sensitivity to *para*-chlorophenylalanine at a minimal inhibitory concentration of 20 mM, supporting the potential application of *AppheS*m as a counterselectable marker in *Av. paragallinarum*. The feasibility of this strategy was evaluated by generating an *ApglgB* insertion mutant harboring the gene *AppheS*m, followed by the seamless integration of the exogenous gene *FtlpxE* into the *ApglgB* locus via the replacement of the previously inserted gene *AppheS*m. This two-step recombination strategy successfully allowed the expression of the integrated gene *FtlpxE*, as assessed by the removal of 1-phosphate groups from lipid A. In addition, the effectiveness of the *pheS*m system in *Av. paragallinarum* was further evaluated by generating marker-free *ApcpsB* and *ApneuB* mutants, which exhibited defects in polysaccharide synthesis and sensitivity to the host complement system. To the best of our knowledge, this is the first study to demonstrate the potential application of *AppheS*m as a counterselectable marker in *Av. Paragallinarum*. Furthermore, the method developed herein is of tremendous importance as a tool for the genetic manipulation of this bacterium.

**IMPORTANCE:**

*Avibacterium paragallinarum* is the etiological agent of infectious coryza, a significant respiratory ailment associated with growth inhibition and diminished egg production in poultry. Given the increasing incidence of antibiotic resistance in bacteria, there is an imperative need for the development of genetically engineered vaccines; however, the lack of effective tools for genetic manipulation in *Av. paragallinarum* has limited the scope of related research. Herein, we investigated the employment of the gene pheSm as a counterselectable marker for the screening of marker-free mutants of *Av. paragallinarum*. We report the successful and precise deletion of multiple genes in *Av. paragallinarum* and an instance of the effective seamless integration of the exogenous gene lpxE using this method. This study presents a valuable genetic tool for investigating the virulence genes and associated mechanisms of pathogenicity of this bacterium, as well as aiding the development of genetically engineered vaccines.

## INTRODUCTION

*Avibacterium paragallinarum* is a gram-negative bacterium that belongs to the family *Pasteurellaceae* and causes infectious coryza (IC), an acute respiratory disease that afflicts chickens (1). The clinical manifestations of IC include nasal congestion, sneezing, dyspnea, facial edema, lacrimation, and diarrhea. Despite widespread and intensive vaccination for the control of the disease, outbreaks of IC continue to persist globally as the vaccine strains often fail to provide cross-protection against the nonmatching field strains (2, 3), leading to considerable economic losses (1, 4).

The genome sequences of several strains of *Av. paragallinarum*, such as the nicotinamide adenine dinucleotide (NAD)-dependent strains 211 and JF4211, nontypical strains AP-2 and AG21-0333, and NAD-hemin-independent strain FARPER-174, have been previously reported (5–8). Further research on the virulence factors of *Av. paragallinarum* is of great importance due to the complexity of its genome and the limited knowledge of the pathogenesis of IC. Capsular polysaccharides (CPS), pilin protein, repeats-in-toxin toxins, membrane vesicles, and phosphorylcholine have been previously proposed as factors associated with the virulence of *Av. paragallinarum* (9–13). The study of virulence factors often employs gene editing, which is a vital method for the engineering of bacterial cells and frequently utilizes homologous recombination–based techniques, such as gene knockout, knockin, and allelic exchange (14). Instances of generation of mutant strains via the employment of gene-editing techniques include the introduction of suicide plasmids in *Riemerella anatipestifer* ATCC11845 via conjugation (15), the introduction of a defective *thy* gene encoding a thymidylate synthase in *Glaesserella parasuis* via natural transformation (16), and the generation of a mutant strain of *Av. paragallinarum* by employing the TargeTron gene knockout system (9). The generation of mutant strains via resistance marker-free mutation has hitherto been accomplished in bacteria of the family *Pasteurellaceae*, exemplified by *Pasteurella multocida* and *R. anatipestifer* (17, 18). However, mutant strains of *Av. paragallinarum* have only been generated based on resistance markers or the introduction of additional intron sequences (9, 19). The disadvantages of such an approach include the retention of the marker on the bacterial genome, which has been previously observed to exert polar effects on distal genes of the mutant operon (20), and the inability to utilize marker-containing mutant strains for the development of vaccines due to potential adverse effects (21). These drawbacks underscore the need to investigate novel gene-editing techniques and to develop simple and efficient gene-manipulation tools for generating marker-free mutants of *Av. Paragallinarum*, thereby facilitating research of its virulence genes and their functions.

A two-step strategy involving selection and counterselection has typically been employed for increasing the efficiency and rate of mutant generation. Counterselectable markers, which are different from positive-selection markers, are effective tools in genetic engineering as they allow selection for loss of genetic markers rather than their presence. Counterselectable markers, such as the genes *rpsL*, *sacB*, *rdxA*, and *pheS*, can be lethal to the host strain under certain conditions (22–24). The gene *pheS*, which encodes the β-subunit of phenylalanyl-tRNA synthetase, is a suitable option for a counterselectable marker, as aminoacyl-tRNA synthetases responsible for charging tRNAs with the same amino acid are typically conserved across species (25). The selection strategy is based on the sensitivity of *Av. paragallinarum* to *para*-chlorophenylalanine (*p*-Cl-Phe), whose incorporation into cellular proteins is lethal to the cells (25, 26). Therefore, this is a precise and efficient method that allows screening for genetic modifications without leaving any trace of foreign DNA. In particular, the gene *pheS* has been exploited for the efficient generation of marker-free mutants, facilitating the study of the molecular mechanisms underlying the pathogenicity and virulence of several bacteria, including *Streptococcus suis*, *R. anatipestifer*, *Listeria monocytogenes*, and *Bacillus amyloliquefaciens* (17, 21, 27, 28); however, an efficient counterselection system for *Av. paragallinarum* has not been developed to date.

The current study showcases the development of a unique and efficient system for allelic exchange that employs a counterselection strategy and is accomplished via two primary steps: first, the initial screening for mutants using erythromycin resistance (Erm^R^) as a marker, followed by the screening for unmarked mutants utilizing the gene *AppheS*m as a counterselectable marker. This approach was employed for achieving targeted knock-out of the genes *ApglgB*, *ApneuB*, and *ApcpsB* in *Av. paragallinarum*. The exogenous gene *FtlpxE* from *Francisella tularensis* encoding lipid A 1-phosphatase was successfully inserted into the *ApglgB* locus and found to be efficiently expressed and functional. This marks the first successful attempt at generating a marker-free deletion mutant of *Av. paragallinarum* and validates the feasibility of applying the counterselectable marker *AppheS*m, generated by introducing a point mutation in the gene *AppheS*, in this bacterium. Furthermore, this method provides a genetic tool for investigating genes associated with the virulence of *Av. paragallinarum*, facilitates the insertion of exogenous genes, and aids the development of genetically modified vaccines.

## RESULTS

### Sensitivity of *Av. paragallinarum* to *p*-Cl-Phe

**Fig 1:**
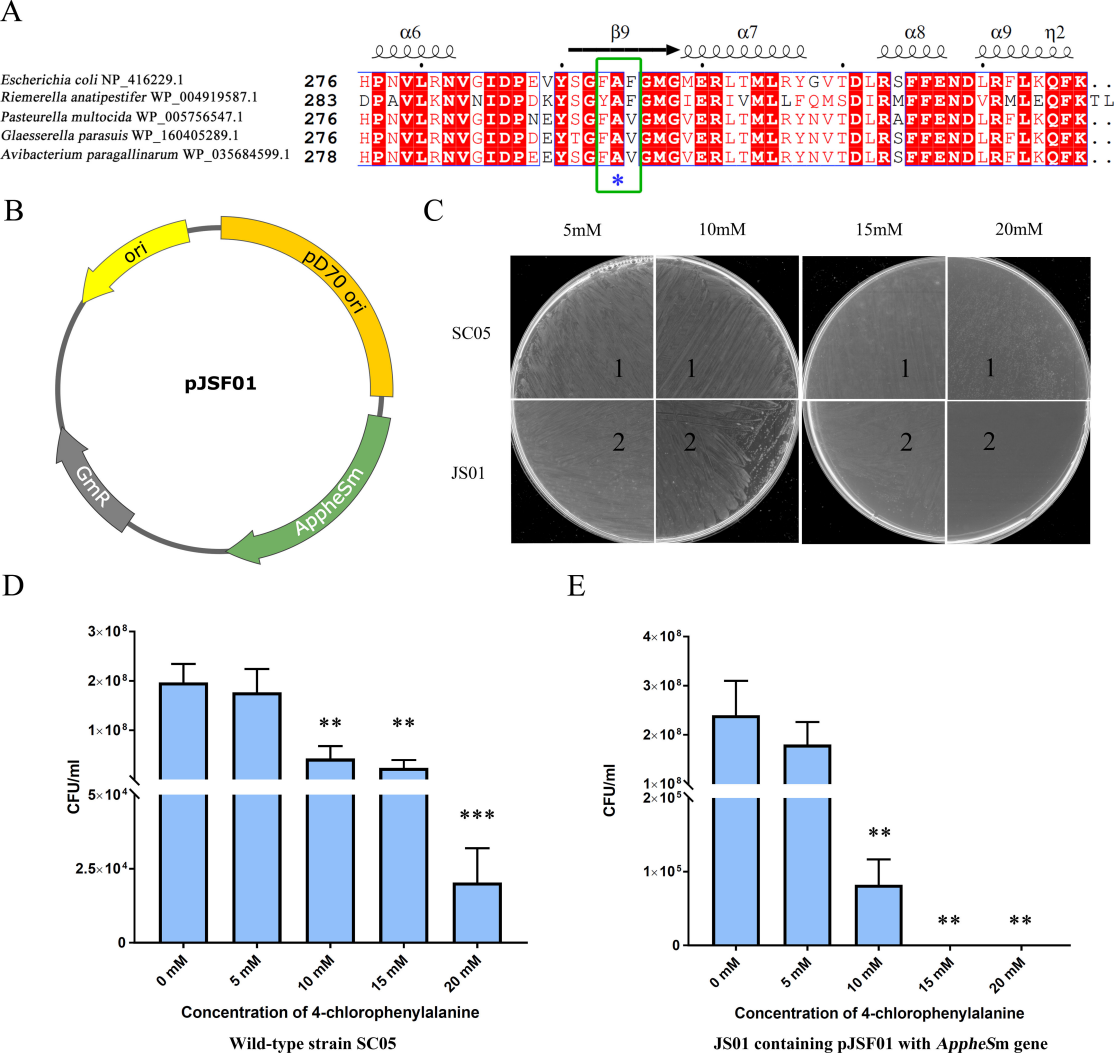
Construction of the *AppheS*m gene and validation of its p-Cl-Phe sensitivity in *Av. paragallinarum*. (**A**) Multiple sequence alignment of *pheS* sequences from *E. coli*, *R. anatipestifer*, *P. multocida*, *G. parasuis*, and *Av. paragallinarum*. Green boxes indicate conserved residues corresponding to alanine at position 294 (A294) of *E. coli pheS*. (**B**) Illustration of the shuttle plasmid pJSF01 containing the gene *AppheS*m. The figure was generated using SnapGene software (GSL Biotech, Chicago, IL, USA). (**C**) Susceptibility of the strains SC05 and JS01 to *p*-Cl-Phe at concentrations of 5, 10, 15, and 20 mM. Survival analysis of SC05 (**D**) and JS01 (**E**) at different *p*-Cl-Phe concentrations. Error bars represent the standard deviation from three independent experiments (*, **, and *** denote *P* < 0.05, *P* < 0.01, and *P* < 0.001, respectively).

Previous studies have demonstrated that bacteria containing a point mutation in the gene *pheS* may exhibit sensitivity to *p*-Cl-Phe (25, 26). Therefore, the gene *pheS* of *Av. paragallinarum* was first explored as a counterselectable marker, and multiple sequence alignment of the gene sequence and those of the corresponding genes from *Escherichia coli*, *R. anatipestifer*, *P. multocida*, and *G. parasuis* was carried out using the program Clustal X. The amino acid alanine at position 294 (A294) of ApPheS was found to be highly conserved across all the selected species (Fig. 1A). Subsequently, a point mutation (A294G) was introduced to generate the mutant gene *AppheS*m, which was cloned into the shuttle plasmid pSF118 to generate the plasmid pJSF01 (Fig. 1B). To validate the feasibility of employing *AppheS*m as a counterselectable marker, the plasmid pJSF01 was introduced into the *Av. paragallinarum* strain SC05 by electroporation to generate the strain JS01. The sensitivity of the strain JS01 to *p*-Cl-Phe was then assessed; heightened sensitivity and significant growth inhibition were observed with increasing concentrations of *p*-Cl-Phe, with total growth inhibition observed at a concentration of 20 mM (Fig. 1C). By contrast, the growth of the parental strain SC05 was not inhibited upon treatment with *p*-Cl-Phe at concentrations of 5, 10, 15, and 20 mM. Survival analysis also showed that at 20 mM *p*-Cl-Phe, the wild-type strain SC05 survived with more than 10⁴ colonies, whereas the *AppheS*m-carrying strain JS01 had no surviving colonies (Fig. 1D and E). Taken together, these results suggest that *AppheS*m can be employed as a counterselectable marker for *Av. paragallinarum* utilizing a *p*-Cl-Phe concentration of 20 mM.

### Construction of a marker-free *ApglgB* mutant of *Av. paragallinarum* utilizing a two-step strategy

**Fig 2:**
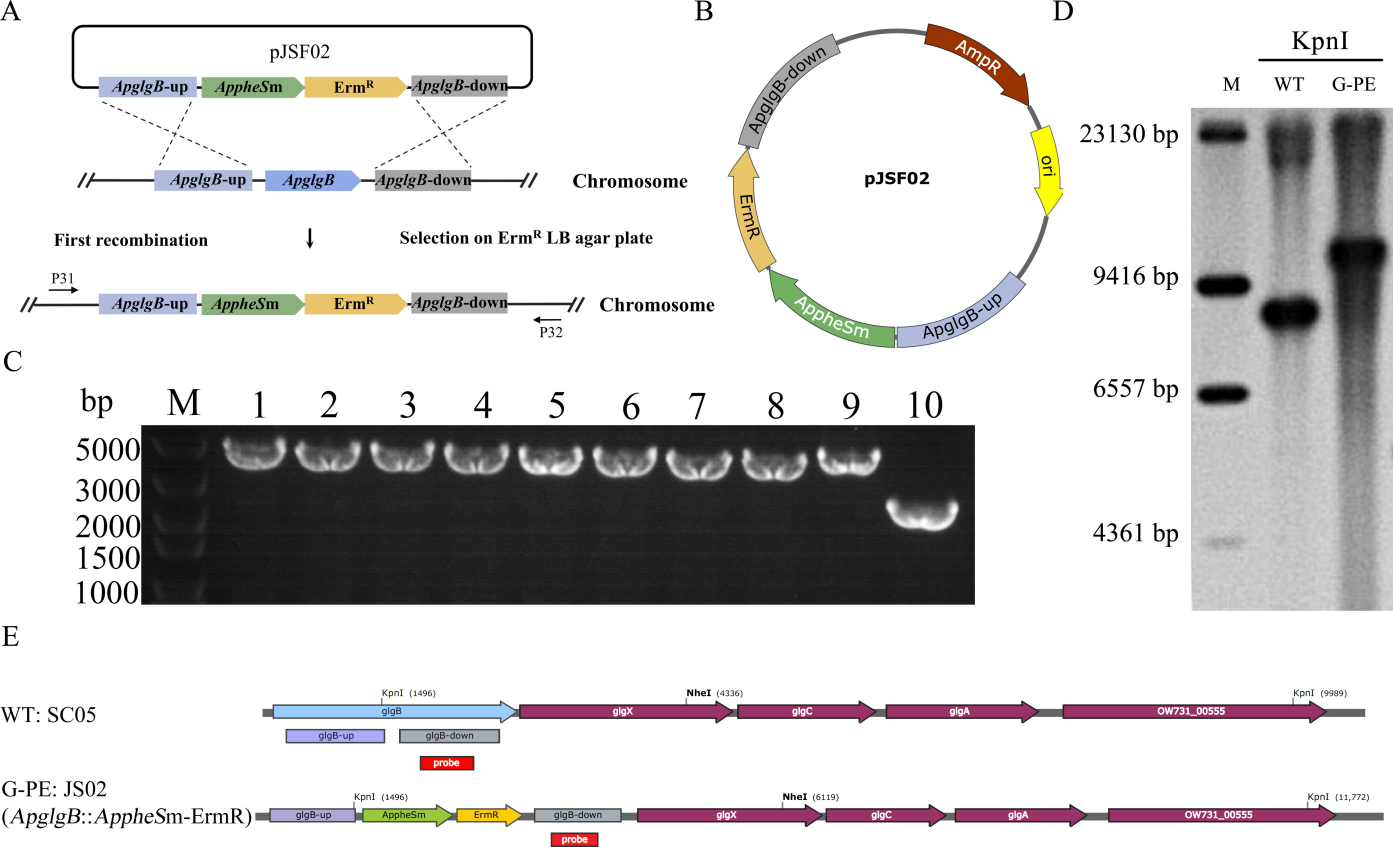
Insertion of the counter-selection marker *AppheS*m into the *ApglgB* locus of *Av. paragallinarum* via natural transformation and homologous recombination. (**A**) Schematic of the first-round natural transformation and subsequent homologous recombination for *AppheS*m cassette integration. (**B**) Structure of suicide plasmid pJSF02 carrying the *ApglgB-AppheS*m knock-out cassette, which includes the genes *AppheS*m and ErmR, together with the upstream and downstream homology arms of *ApglgB*. (**C**) Colony PCR analysis and agarose gel electrophoresis of transformant strain JS02 generated via homologous recombination. Lane M: DL5000 DNA marker. Lanes 1–9: Amplicons (4,411 bp) obtained via colony PCR from the JS02 transformants using the primer pair P31/P32. Lane 10: Amplicon (2,536 bp) obtained from the wild-type strain SC05 (control). (**D**) Southern blot of KpnI-digested genomic DNA from the *ApglgB* insertion mutant with the *AppheS*m cassette. Lanes: M, DIG-labeled marker; WT, wild-type strain SC05; G-PE, *ApglgB::AppheS*m-Erm^R^. (**E**) Genomic organization adjacent to *ApglgB* and distribution of restriction sites for Southern blot analysis. WT, wild-type strain SC05; G-PE: JS02, *glgB* disrupted by *AppheS*m and Erm^R^.

The strategy involves the insertion of the markers *AppheS*m and Erm^R^ into the *ApglgB* locus via natural transformation, as illustrated in Fig. 2A. The plasmid pJSF02 containing the *ApglgB-AppheS*m knock-out cassette (Fig. 2B) was generated and employed for the first natural transformation of the strain SC05 followed by homologous recombination to generate the *ApglgB* insertion mutant (strain JS02); this mutant was cultivated on Luria–Bertani (LB) agar plates supplemented with erythromycin to facilitate screening. Single colonies were subsequently selected for analysis via polymerase chain reaction (PCR) using the primer pair P31/P32; a successful knock-out yielded an amplicon of 4,411 bp, and the positive control SC05 yielded an amplicon of 2,536 bp. A total of 30 colonies were analyzed, and representative results are presented in Fig. 2C. The *ApglgB* mutant was further confirmed by Southern blot (Fig. 2D and E; WT, wild-type strain SC05; G-PE, *ApglgB::AppheS*m-Erm^R^), and details are provided in the Methods and Materials section. The analysis allowed the identification of the strain JS02 containing an insertion of the markers *AppheS*m and Erm^R^ at the *ApglgB* locus; this strain was subsequently employed for a second round of natural transformation.

**Fig 3:**
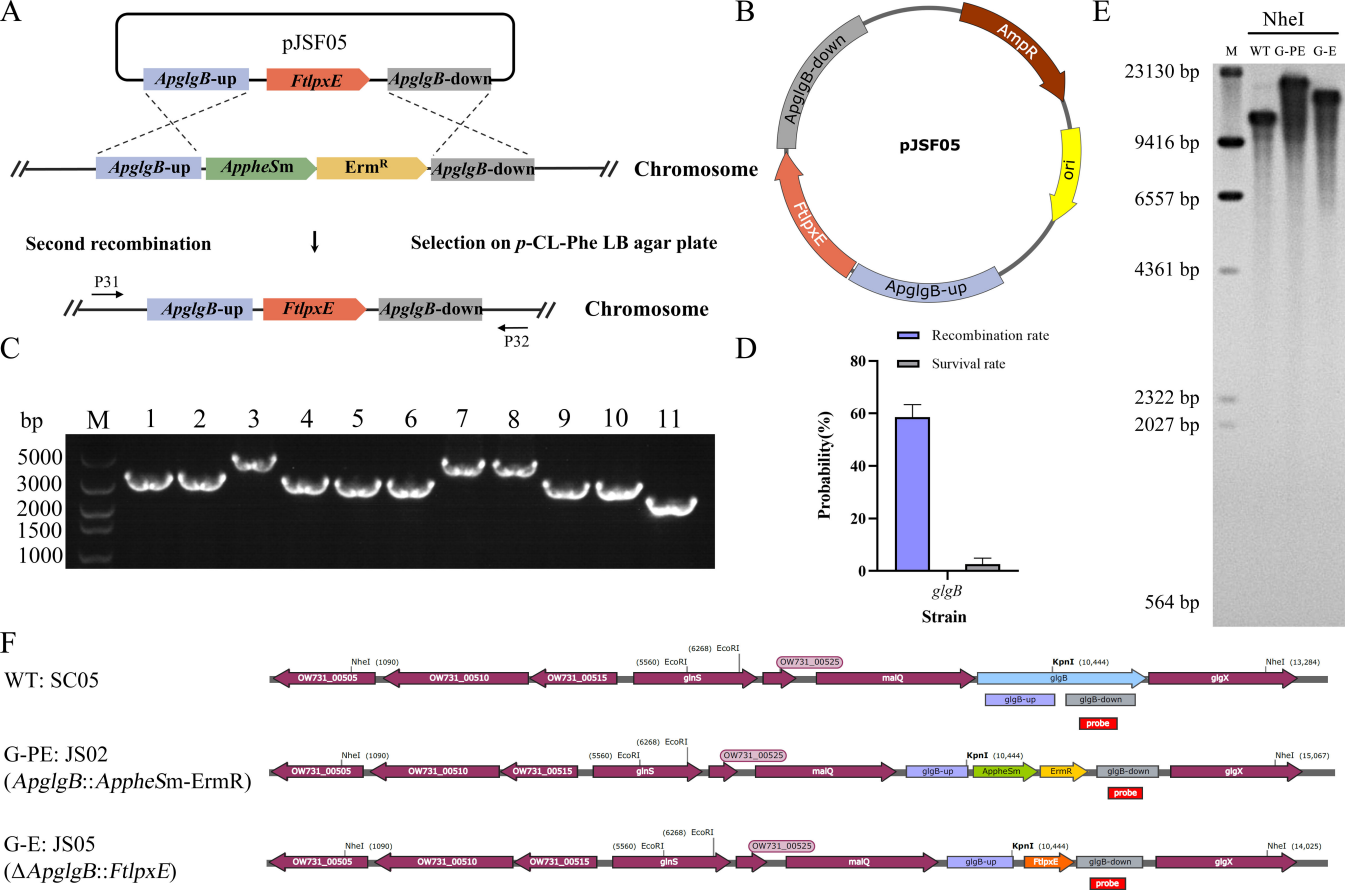
Generation of a marker-free *ApglgB* mutant of *Av. paragallinarum* via the second round of natural transformation. (**A**) Schematic diagram of the second-round natural transformation for the genomic integration of *FtlpxE*. (**B**) Suicide plasmid pJSF05 containing the *ApglgB::FtlpxE*-markerless cassette that includes the gene *FtlpxE* as well as upstream and downstream homology arms of *ApglgB*. (**C**) Colony PCR and agarose gel electrophoresis of JS05 transformants obtained from the second natural transformation. Lane M: DL5000 DNA marker. Lanes 1–10: Amplicons obtained via colony PCR from the marker-free mutant strain JS05 (3,208 bp) or the strain JS02 (4,411 bp), respectively, using the primer pair P31/P32. The wild-type strain SC05 was employed as a control (line 11). (**D**) Survival and screening rates of marker-free *ApglgB* deletion transformants via the *AppheS*m system. Survival rate = (number of colonies growing on the LB agar plate containing *p*-Cl-Phe/total number of colonies growing on LB agar plate) × 100; Recombination rate = (number of positive colonies/total number of colonies growing on the LB agar plate containing *p*-Cl-Phe) × 100. (**E**) Southern blot analysis of NheI-digested genomic DNA from the *ApglgB::FtlpxE* insertion mutant. Lanes: M, DIG-labeled marker; WT, wild-type strain SC05; G-PE, *ApglgB::AppheS*m-Erm^R^; G-E, *ApglgB::FtlpxE*. (**F**) Schematic representation of the genomic structure of the *FtlpxE* integration strain for Southern blot analysis. WT, wild-type strain SC05; G-PE: JS02, *ApglgB* disrupted by *AppheS*m and Erm^R^; G-E: JS05, *ApglgB* insertion by *FtlpxE*.

In order to achieve the removal of resistance markers from the mutant strain, a cassette containing the *ApglgB* locus with an insertion of the gene *FtlpxE* (*ApglgB::FtlpxE*) and lacking any resistance marker was constructed for counterselection. The insertion mutant was identified by screening colonies obtained on LB agar plates containing 20 mM p-Cl-*P*he (Fig. 3A). Toward this end, the plasmid pJSF05 (Fig. 3B) containing the *ApglgB::FtlpxE*-markerless knock-in cassette was employed for the natural transformation of the strain JS02 to allow the second recombination event. PCR analysis using the primer pair P31/P32 yielded an amplicon of 3,208 bp for single-colony transformants (strain JS05) compared with 4,411 bp otherwise, while the wild-type control SC05 yielded an amplicon of 2,536 bp, as mentioned previously. A total of 30 colonies were analyzed, and representative results are presented in Fig. 3C. Southern blot analysis was performed to further confirm the *FtlpxE*-integrated strain (Fig. 3E and F; G-PE, *ApglgB::AppheS*m-Erm^R^; G-E, *ApglgB::FtlpxE*). The efficiency of screening for the *ApglgB* mutant strain using the *AppheS*m system was then determined. The number of colonies of the transformants on LB plates lacking and containing 20 mM *p*-Cl-Phe was found to be approximately 10^3^ and 3 × 10 CFU, respectively, corresponding to a survival rate and an estimated recombination efficiency of approximately 3.63% ± 3.23% and 58.57% ± 7.08%, respectively (Fig. 3D). The successful generation of the *ApglgB::FtlpxE* mutant (strain JS05) proves that the *AppheS*m system can be employed for achieving marker-free gene knock-in in *Av. paragallinarum*.

### Integration of *FtlpxE* allows the effective removal of a phosphate group from lipid A in *Av. paragallinarum*

**Fig 4:**
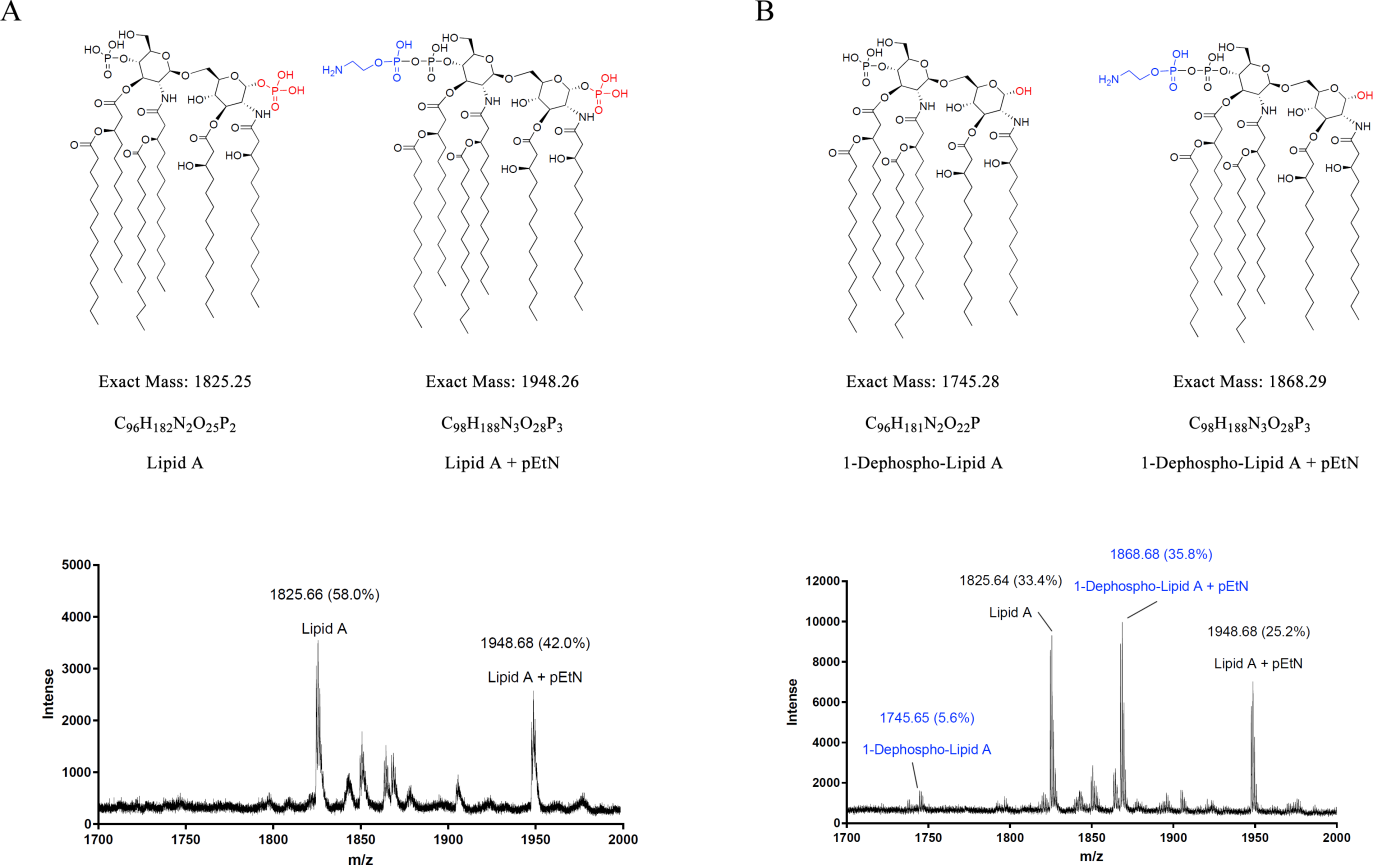
MALDI-TOF MS analysis of lipid A extracted from *Av. paragallinarum* strains SC05 (**A**) and JS05 (**B**), with predicted structures of the detected [M–H]⁻ ions.

Lipid A, also known as endotoxin, is essential for the survival of almost all gram-negative bacteria and can induce inflammation as well as fever as an immune response in higher organisms (29). The lactone phosphatase FtLpxE from *Francisella novicida* is known to eliminate 1-phosphate groups from lipid A at the external surface of the inner membrane (30). Herein, samples of lipid A isolated from the wild-type (SC05) and *ApglgB::AplpxE* (JS05) strains of *Av. paragallinarum* were individually combined with a 2,5-dihydroxybenzoic acid matrix. The resulting mixture was subjected to matrix-assisted laser desorption ionization-time of flight mass spectrometry (MALDI-TOF MS) analysis in negative-ionization mode, as previously described (31). As shown in Fig. 4, two different lipid A structures are hereby identified in *Av. paragallinarum* SC05 (Fig. 4A). The unmodified lipid A with m/z 1825.66 was highly abundant (58.0%), while the other compound, with m/z 1948.68 and an abundance of 42.0%, was speculated to be an acetamide phosphate (pEtN)-modified derivative of lipid A based on its molecular weight. As anticipated, the expression of FtLpxE in the strain SC05 resulted in the production of a significant quantity of lipid A dephosphorylated at the 1-position (Fig. 4B). This yielded compounds with m/z values of 1,745.65 (abundance of 5.6%) and 1,868.68 (35.8%), corresponding to a decrease of 80 in the m/z values of the compounds lipid A and pEtN-modified lipid A, respectively. These results show that the integration of *FtlpxE* via the two-step homologous recombination strategy employed herein, and its subsequent expression of the gene, resulted in the loss of the phosphate group from a large population of lipid A molecules. This indicates that the marker *AppheS*m can be employed for achieving seamless integration of exogenous genes in *Av. paragallinarum*, with the integrated gene *FtlpxE* allowing an efficient modification of lipid A in this bacterium.

### *AppheS*m-mediated insertion of tag sequence into target gene

**Fig 5:**
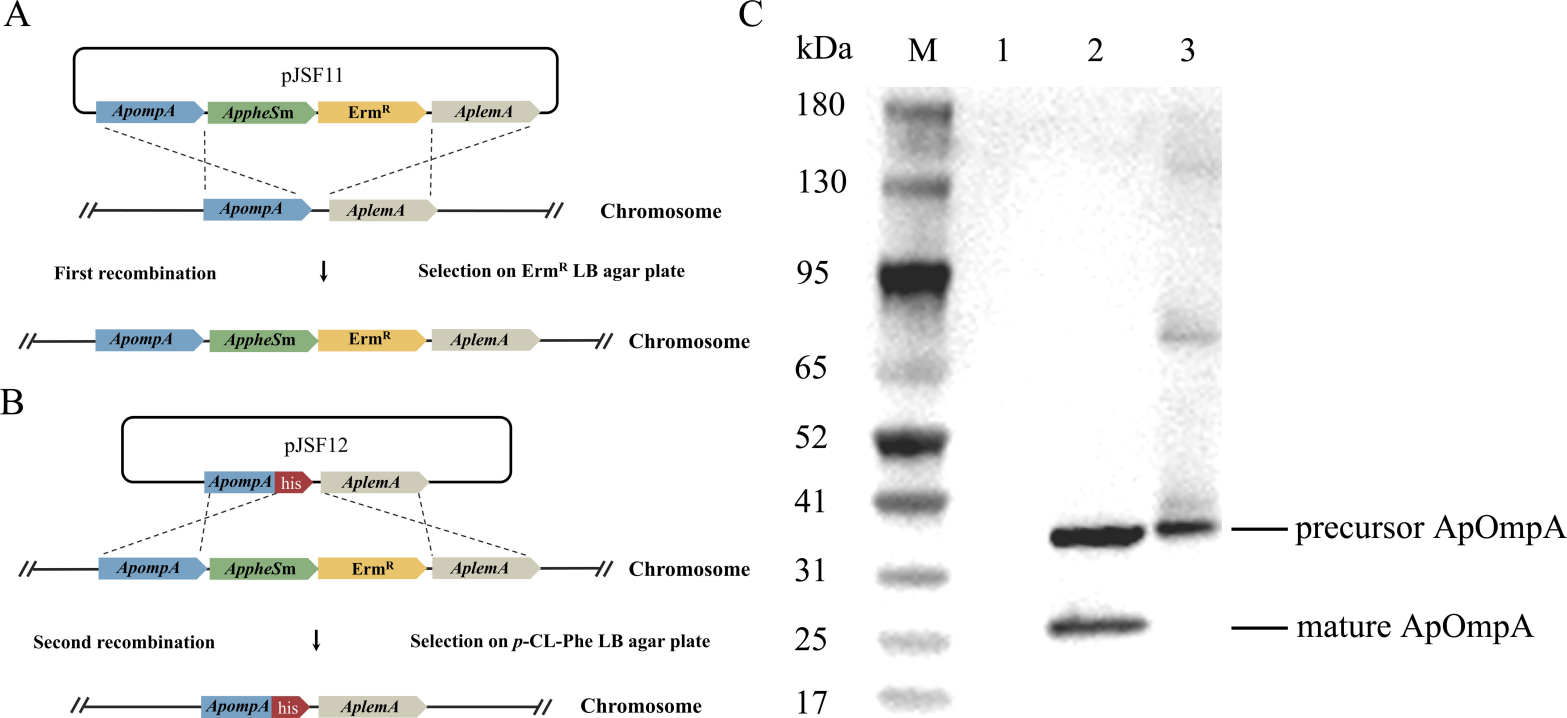
Construction of an *Av. paragallinarum* strain expressing C-terminally His-tagged ApOmpA. (**A**) Schematic representation of the first round of natural transformation at the *ApompA* locus. (**B**) Illustration of homologous recombination for His-tag introduction during the second natural transformation at the *ApompA* locus. (**C**) Western blot analysis of the His-tagged *ApompA* strain. Lanes: M, marker; 1, wild-type strain SC05; 2, His-tagged *ApompA* strain; 3, positive control, ApOmpA with His-tag expressed and purified from *E. coli*.

To further demonstrate that the *AppheS*m system can introduce tag sequences into specific genes, *ApompA* was selected as the target gene for His-tag insertion. First, using the plasmid pJSF11, the *AppheSm* cassette with an antibiotic resistance marker was inserted at the 3′ end of the *ApompA* gene (Fig. 5A). Subsequently, the *AppheS*m cassette in the chromosome was replaced with the integration plasmid pJSF12 carrying *ApompA*-His-tag, resulting in a recombinant strain containing the His tag but lacking any antibiotic resistance marker (Fig. 5B). Western blot analysis of the recombinant strain revealed two bands at approximately 31 kDa and 25 kDa, corresponding to the precursor and the mature form of ApOmpA, respectively (Fig. 5C, lane 2). In contrast, no signal was detected in the wild-type strain due to the absence of the His tag (lane 1). These results indicate that the *AppheS*m system enables efficient introduction of tag sequences into target genes, thereby providing a useful tool for functional gene studies.

### Construction of marker-free *ApcpsB* and *ApneuB* mutants of *Av. paragallinarum* utilizing the *AppheS*m system

**Fig 6:**
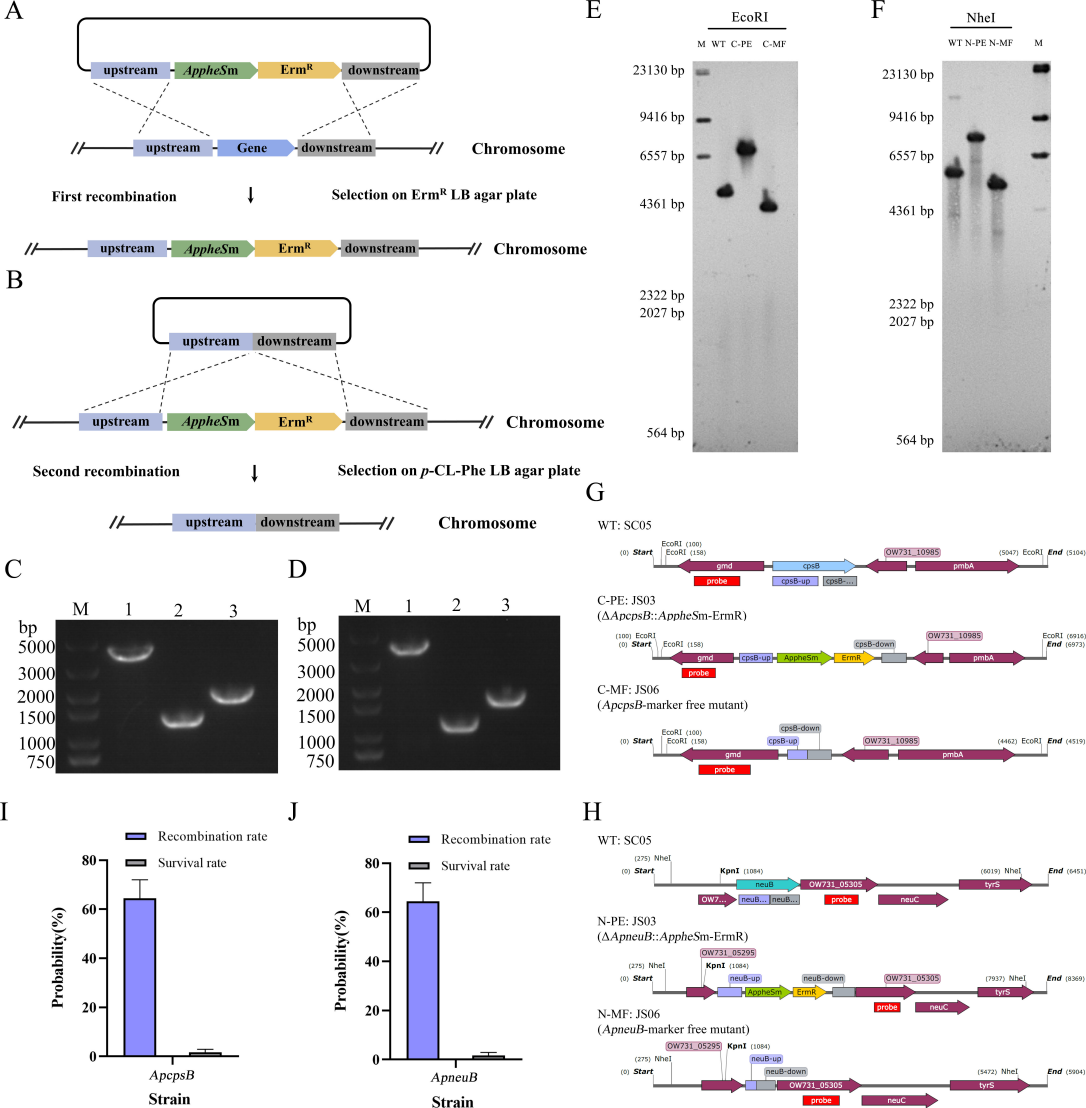
Generation and validation of marker-free *ApcpsB* and *ApneuB* mutants of *Av. paragallinarum* using a two-step recombination strategy (**A**) The first-round homologous recombination by natural transformation integrates the *AppheS*m cassette into the target gene locus. (**B**) The second homologous recombination, performed via a subsequent natural transformation, excises the *AppheS*m cassette to yield a marker-free mutant. (**C**) Colony PCR analysis and agarose gel electrophoresis for the verification of transformants. Lane M: DL5000 DNA marker. Lane 1: Strain JS03 (amplicon of size 3,908 bp). Lane 2: Strain JS06 (1,308 bp). Lane 3: Strain SC05 (1,849 bp). (**D**) Colony PCR analysis and agarose gel electrophoresis for the verification of transformants. Lane M: DL5000 DNA marker. Lane 1: Strain JS04 (amplicon of size 3,745 bp). Lane 2: Strain JS07 (1,134 bp). Lane 3: Strain SC05 (1,645 bp). (**E**) Southern blot analysis of EcoRI-digested genomic DNA from the *ApcpsB* markerless mutant. Lanes: M, DIG-labeled marker; WT, wild-type strain SC05; C-PE, *ApcpsB::AppheS*m-Erm^R^; C-MF, *ApcpsB* markerless mutant. (**F**) Southern blot analysis of NheI-digested genomic DNA from the *ApneuB* markerless mutant. Lanes: M, DIG-labeled marker; WT, wild-type strain SC05; N-PE, *ApneuB::AppheS*m-Erm^R^; N-MF, *ApneuB* markerless mutant. (**G**) Genomic structure of *ApcpsB* and distribution of restriction sites for Southern blot analysis. WT, wild-type strain SC05; C-PE: JS03, *ApcpsB* disrupted by *AppheS*m and Erm^R^; C-MF: JS06, *ApcpsB* marker-free mutant. (**H**) Genomic structure of *ApneuB* and distribution of restriction sites for Southern blot analysis. WT, wild-type strain SC05; N-PE: JS04, *ApneuB* disrupted by *AppheS*m and Erm^R^; N-MF: JS07, *ApneuB* marker-free mutant. (**I**) Survival rate and screening efficiency of transformants with marker-free deletion of *ApcpsB* generated using the *AppheS*m system. (**J**) Survival rate and screening efficiency of transformants harboring a marker-free deletion of *ApneuB* constructed via the *AppheS*m system. The formula for the rates of survival and recombination has been described in the legends to Fig. 3.

The seamless, successful, and effective integration of the exogenous gene *FtlpxE* in *Av. paragallinarum* using the counterselectable marker *AppheS*m has been confirmed herein. The efficacy of the *pheS*m system in *Av. paragallinarum* was further validated by carrying out a seamless knock-out of two genes *ApcpsB* and *ApneuB*, which are associated with polysaccharide synthesis. A successful knock-out of the genes *ApcpsB* and *ApneuB* via the two-step recombination approach was validated by PCR analysis, which revealed that the corresponding amplicons differed in size from those of the strain SC05 by approximately 500 bp each; the illustration of the procedure is presented in Fig. 6A and B. The plasmids pJSF03, pJSF04, pJSF06, and pJSF07 containing the *ApcpsB::AppheS*m, *ApneuB::AppheS*m, *ApcpsB-*marker-free, and *ApneuB-*marker-free knock-out cassettes, respectively, were employed for the generation of marker-free deletion mutants of *ApcpsB* and *ApneuB*.

The *ApcpsB* mutant strain was generated by employing the plasmid pJSF03 (containing the *ApcpsB::AppheS*m knock-out cassette) for the first natural transformation of the strain SC05, followed by selection of the transformants on LB agar plates containing erythromycin and screening using colony PCR. An amplicon size of 3,908 bp (Fig. 6C, lane 1) reflected successful transformation to yield the mutant *ApcpsB::AppheS*m (strain JS03), which was subjected to sequencing analysis for further verification (data not shown). The genome of the strain JS03 carries the gene encoding Erm^R^, and its removal was accomplished via the second natural transformation of the strain JS03 with the plasmid pJSF06 containing the *ApcpsB-*marker-free knock-out cassette. The resulting transformants were cultured on LB agar plates supplemented with 20 mM *p*-Cl-Phe and subjected to colony PCR analysis, with fragment sizes of 1,308 and 1,849 bp corresponding to the mutant and wild-type strains, respectively (Fig. 6C. lanes 2 and 3). The mutant strain obtained (strain JS06) was subjected to sequencing and Southern blot analysis for further verification (Fig. 6E and G; C-PE, *ApcpsB::AppheS*m-Erm^R^; C-MF, *ApcpsB* markerless mutant). The *ApneuB* mutant strain was generated by employing the plasmid pJSF04 (containing the *ApneuB::AppheS*m knock-out cassette) for the first natural transformation of the strain SC05 and selection on LB agar plates containing erythromycin, as mentioned previously. The transformants were verified by colony PCR (yielding an amplicon size of 3,745 bp; Fig. 6D, lane 1) and subjected to sequencing analysis to yield the mutant *ApneuB::AppheS*m (strain JS04). Similar to the strain JS03, the strain JS04 also carries the gene encoding Erm^R^, whose removal was accomplished via the second natural transformation of the strain JS04 with the plasmid pJSF07 (containing *ApneuB-*marker-free knock-out cassette), selection of the transformants on LB agar plates supplemented with 20 mM *p*-Cl-Phe, and screening via colony PCR and sequencing; amplicons of sizes 1,134 and 1,645 bp were obtained from the mutant and wild-type strains, respectively (Fig. 6D lanes 2 and 3). The resulting mutant strain JS07 was subjected to sequencing and Southern blot analysis for verification (Fig. 6F and H; N-PE, *ApneuB::AppheS*m-Erm^R^; N-MF, *ApneuB* markerless mutant). A comparison of the numbers of bacterial colonies obtained on LB agar plates without and with 20 mM *p*-Cl-Phe (10^3^ and 3 × 10 CFU, respectively) revealed that the efficiency of recombination at the *ApcpsB* locus using the *AppheS*m system was 70.11% ± 5.97%, while the survival rate was 3.44% ± 2.07% (Fig. 6I). The efficiency of recombination in the marker-free *ApneuB* mutant strains generated using the *AppheS*m system was 63.87% ± 7.05%, while the survival rate was 2.91% ± 2.05% (Fig. 6J). These results indicate that the counterselectable marker *AppheS*m can be effectively employed for the generation of seamless knock-out of multiple genes in *Av. paragallinarum*.

### Phenotypic analysis of the *ApcpsB* and *ApneuB* mutant strains

**Fig 7:**
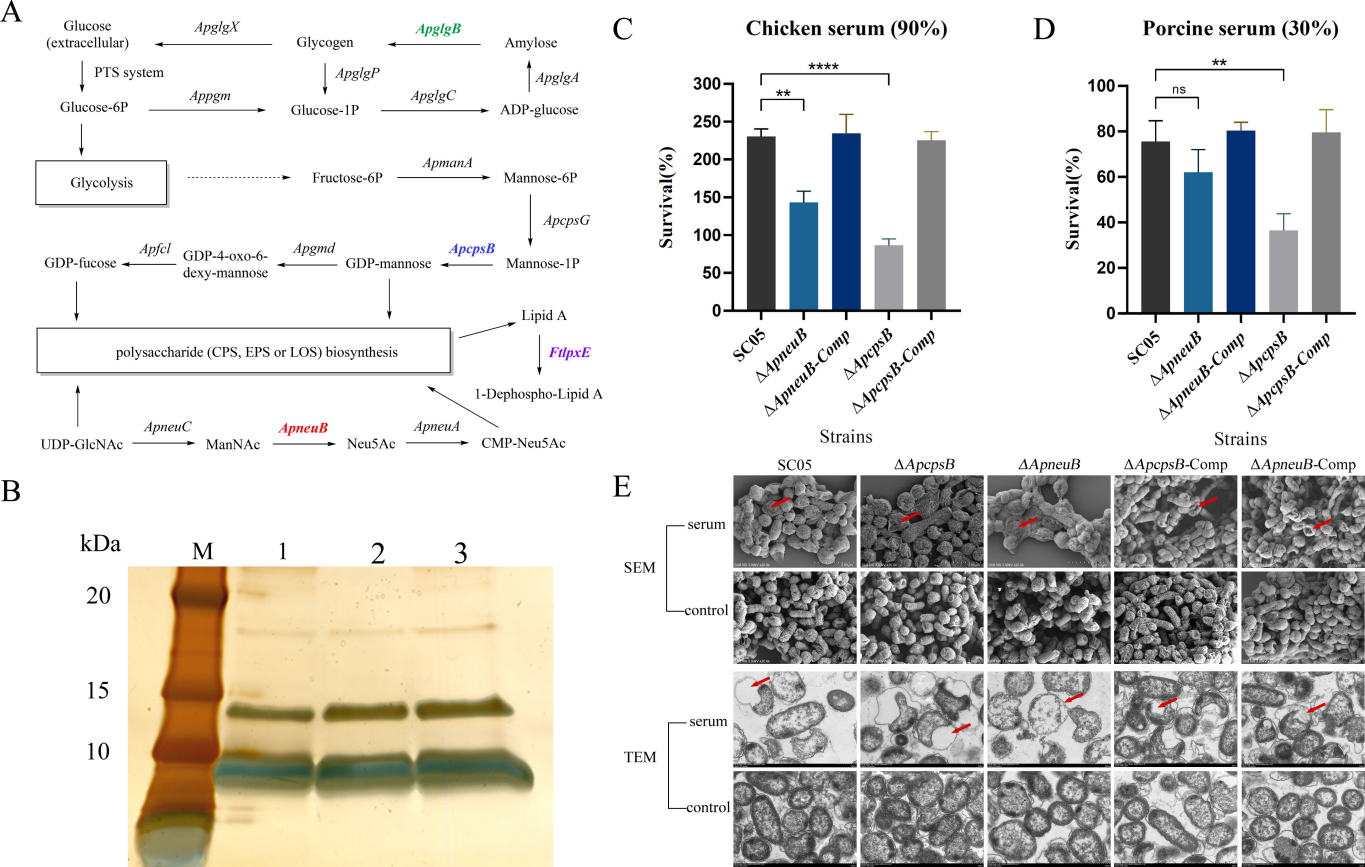
Phenotypic characterization of *ApcpsB* and *ApneuB* mutant strains of *Av. paragallinarum*. (**A**) Schematic depicting the metabolic synthesis pathway of polysaccharides and the roles of the genes *ApcpsB* and *ApneuB*. (**B**) LOS profiles of the wild-type SC05 and *ApcpsB* as well as *ApneuB* mutant strains assessed using 15% SDS–PAGE. Lane M: Protein molecular weight marker. Lane 1: Strain SC05. Lane 2: *ApcpsB* mutant strain. Lane 3: *ApneuB* mutant strain. Survival of *Av. paragallinarum* strains in 90% chicken (**C**) and 30% porcine (**D**) sera. The survival percentage was calculated as the ratio of colonies in fresh serum to those in heat-treated serum. Error bars represent the standard deviation from three independent experiments (*, **, ***, and **** denote *P* < 0.05, *P* < 0.01, *P* < 0.001, and *P* < 0.0001, respectively). (**E**) Scanning and transmission electron microscopy analysis of *ApcpsB* and *ApneuB* mutant strains. Red arrows exhibited wrinkling, collapsed, and lysed structures on the cell membrane.

The genes *ApcpsB* and *ApneuB* were predicted to encode mannose-1-phosphate guanylyltransferase and N-acetylneuraminate synthase, respectively, and may play a role in the synthesis of lipooligosaccharides (LOS) or capsular polysaccharides (CPS) (Fig. 7A). The alterations in the content of LOS in the *ApcpsB* and *ApneuB* mutant strains with respect to that in the wild-type strain SC05 were detected by gradient sodium dodecyl sulfate-polyacrylamide gel electrophoresis (SDS–PAGE) followed by silver staining. As shown in Fig. 7B, the rate of migration of the LOS from these three strains is nearly identical, corresponding to molecular weights of approximately 15–18 kDa, indicating that the genes *ApneuB* and *ApcpsB* do not influence the glycoforms of LOS in *Av. paragallinarum* SC05. Due to the absence of monoclonal antibodies against CPS, CPS detection was not performed.

The roles of *ApneuB* and *ApcpsB* in serum resistance were evaluated by assessing the sensitivity of the *ApneuB* and *ApcpsB* mutants, as well as the corresponding complemented strains, to 90% chicken or 30% porcine sera. The survival rate of the wild-type strain SC05 in the presence of 90% chicken serum was found to be 230.33% ± 10.01%, with the *ApneuB* mutant, *ApneuB-*complemented mutant, *ApcpsB* mutant, and *ApcpsB-*complemented mutant strains exhibiting survival rates of 142.67% ± 15.01%, 234.74% ± 11.36%, 86.54% ± 8.5%, and 225.84% ± 11.68%, respectively (Fig. 7C). These observations reveal that the sensitivity of the *ApneuB* mutant strain to chicken serum was significantly higher than that of the wild-type strain SC05 (*P* < 0.01), as was that of the *ApcpsB* mutant strain (*P* < 0.0001); however, the complementation of these mutant strains with the corresponding genes (*ApneuB* and *ApcpsB*, respectively) restored their serum-resistance phenotype.

As shown in Fig. 7D, the survival rate of the wild-type strain SC05 in the presence of 30% porcine serum was found to be 75.56% ± 9.16%, while those of the *ApneuB* mutant, *ApneuB-*complemented mutant, *ApcpsB* mutant, and *ApcpsB-*complemented mutant strains were found to be 61.59% ± 9.53%, 80.34% ± 8.73%, 36.58% ± 7.28%, and 79.51% ± 10.13%, respectively. Importantly, the sensitivity of the *ApneuB* mutant strain to porcine serum, unlike its sensitivity to chicken serum, was not significantly different from that of the wild-type strain SC05 (*P* > 0.05); however, the sensitivity of the *ApcpsB* mutant strain remained significantly different (*P* < 0.01). The complementation of these mutant strains with the corresponding genes (*ApneuB* and *ApcpsB*, respectively) effectively restored their serum-resistance phenotype. Furthermore, LOS extracted from complemented strains were subjected to SDS-PAGE analysis; however, no obvious differences in LOS profiles were observed (Fig. S1). Scanning electron microscopy (SEM) and transmission electron microscopy (TEM) analyses revealed that the *ApcpsB* and *ApneuB* deletion mutants exhibited more pronounced structural damage under the action of complement (Fig. 7E). These results demonstrate that both *ApcpsB* and *ApneuB* play roles in protecting *Av. paragallinarum* against complement-mediated killing.

**Fig 8:**
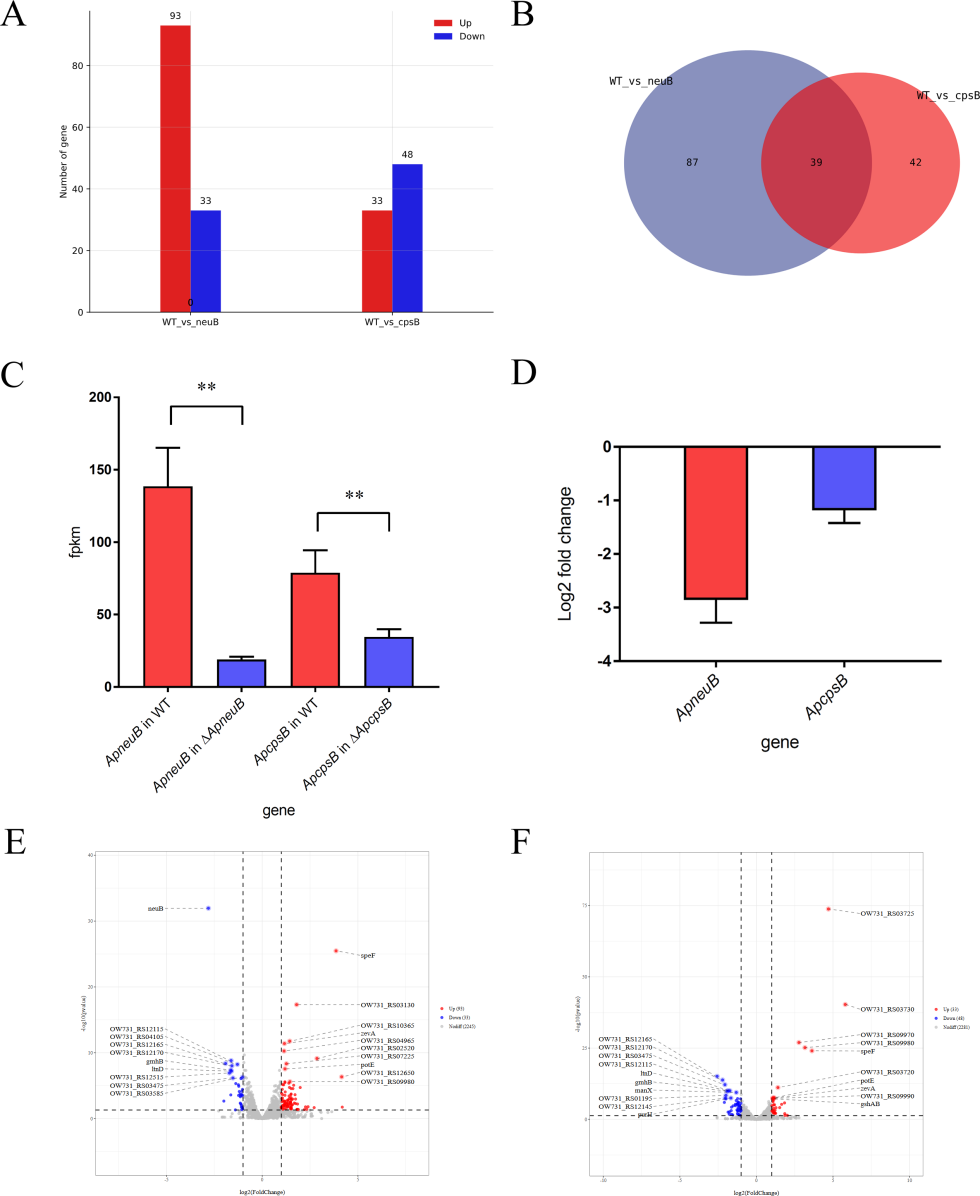
Comparative transcriptomic analysis of *ApcpsB* and *ApneuB* mutant strains of *Av. paragallinarum*. (**A**) Statistics of differentially expressed genes (DEGs) in *ApneuB* and *ApcpsB* mutant strains. (**B**) Shared DEGs between *ApneuB* and *ApcpsB* mutant strains. (**C**) The fragments per Kilobase of transcript per Million mapped reads values (FPKM) of *ApneuB* and *ApcpsB* calculated from RNA-seq data. (**D**) Changes in transcript levels of *ApneuB* and *ApcpsB* genes in their respective mutant strains. Volcano plot analysis of differentially expressed genes in *ApneuB* (**E**) and *ApcpsB* (**F**) mutant strains.

Transcriptomic analysis revealed that, relative to the wild-type strain, the *ApneuB* mutant exhibited 93 upregulated and 33 downregulated genes, whereas the *ApcpsB* mutant displayed 33 upregulated and 48 downregulated genes (Fig. 8A). A total of 39 genes showed differential expression in both mutants (Fig. 8B). The transcriptome data further indicated that deletion of *ApneuB* or *ApcpsB* led to a marked reduction in the fragments per kilobase of transcript per million mapped reads (FPKM) values for both genes, which can be attributed to the removal of several hundred base pairs from each locus, thereby reducing the number of mapped reads (Fig. 8C). This observation provides additional confirmation that the intended genetic edits were successfully introduced. The transcript level of *ApneuB* decreased 7.06-fold, while *ApcpsB* decreased 2.27-fold (Fig. 8D). In the *ApneuB* mutant, significantly upregulated genes included *speF*, OW731_R03130 (S6 family peptidase), OW731_R02520 (YcgL domain-containing protein), *zevA*, and *potE*, among which *speF* and *potE* are both involved in ornithine metabolism. Conversely, significantly downregulated genes included *ApneuB*, *gmhB*, *ltnD*, OW731_R12170 (Mu transposase C-terminal domain-containing protein), and OW731_R04105 (aromatic amino acid transporter), reflecting the altered transcriptional level of *ApneuB* (Fig. 8E). In the *ApcpsB* mutant, significantly upregulated genes included OW731_R03725 (Sel1 repeat family protein), OW731_09970 (class I S-adenosylmethionine-dependent DNA methyltransferase), *speF*, and OW731_R03720. Downregulated genes included OW731_R12165 (AAA family ATPase), OW731_R12170 (Mu transposase C-terminal domain-containing protein), OW731_R03475 (*csrA*), and *manX*, suggesting potential impacts on carbon source metabolism (Fig. 8F).

**Fig 9:**
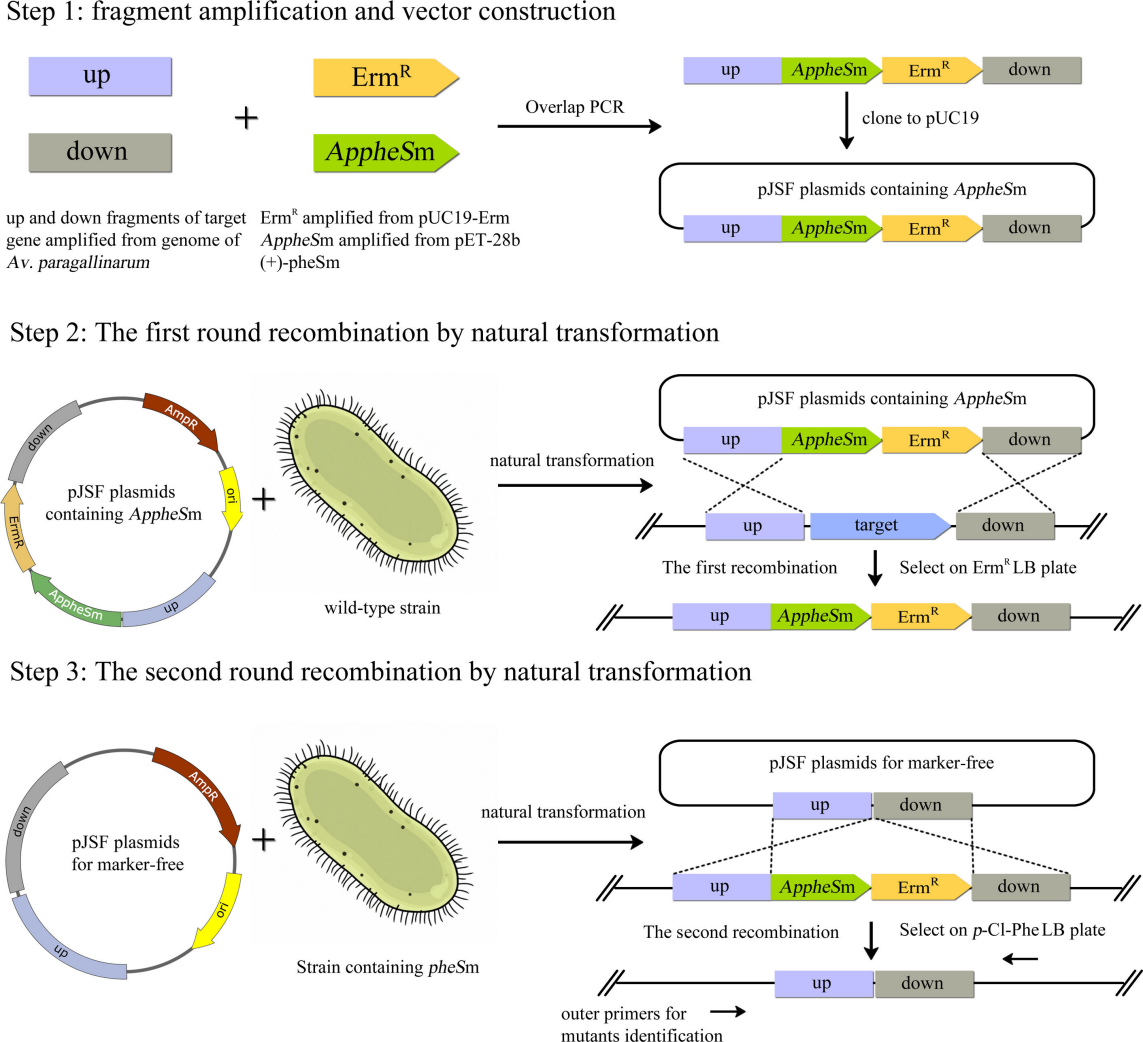
Schematic workflow for markerless *Av. paragallinarum* strain construction.

In conclusion, we have developed an *AppheS*m-based genetic modification approach for *Av. paragallinarum*, with the overall workflow summarized in Fig. 9. This method provides a technical foundation for the investigation of virulence-associated genes and the development of vaccines for this pathogen.

## DISCUSSION

Despite the worldwide prevalence of IC caused by *Av. paragallinarum*, the pathogenesis of the disease remains poorly understood compared to that of diseases caused by other pathogenic bacteria, mainly due to the absence of effective and exhaustive tools for gene manipulation. Although the feasibility of applying several methods of constructing mutant strains has been demonstrated in *Av. paragallinarum* (9, 19, 32, 33), there remains a lack of effective tools for marker-free and scarless gene editing. To address this shortcoming, a counterselection method was additionally employed herein to develop a scarless gene-editing approach of higher efficacy. The feasibility of applying counterselection methods has been previously demonstrated in *P. multocida* and *R. anatipestifer* (34, 35), which are closely related to *Av. paragallinarum*, prompting us to investigate the potential application of *AppheS*m as a counterselectable marker in *Av. paragallinarum*. Toward this end, the growth of wild-type *Av. paragallinarum* in *p*-Cl-Phe was first assessed, given the inability of a majority of bacteria of the family *Sphingomonadaceae* to grow in the presence of 0.1 mM *p*-Cl-Phe; such bacteria are, therefore, not amenable to the employment of *pheS* as a counterselectable marker (36). Additionally, the host bacterium containing *pheS*m must be susceptible to *p*-Cl-Phe to enable successful counterselection. Previous studies have demonstrated that *R. anatipestifer*, *P. multocida*, and *Lactococcus lactis* are susceptible to *p*-Cl-Phe at concentrations of 13 mM (17), 20 mM (34), and 15 mM (37), respectively. The optimal concentration of *p*-Cl-Phe for performing counterselection in *Av. paragallinarum* was experimentally determined herein as 20 mM, indicating that the gene *AppheS*m confers relatively less sensitivity to *p*-Cl-Phe than other bacteria mentioned above, but can still be employed for screening. Subsequently, a two-step strategy for modifying target genes in *Av. paragallinarum* using the *AppheS*m system was devised herein.

Screening efficiency is an important consideration in the construction of mutant strains. While the screening efficiency is obviously influenced by the target gene involved, the hallmark of an ideal screening system is its ability to facilitate the efficient knock-out of multiple different genes. For example, screening efficiencies of 50% and 30% were obtained during the generation of mutants of *L. lactis* containing marker-free knock-out of the galactose operon and the gene *aldB*, respectively, using the *pheS*m counterselectable marker system (37). The efficiency of employing the *pheS*m system for the screening of *glgB*, *hyaE*, *fur*, and *opa* mutants of *P. multocida* was 50.0%, 39.6%, 31.3%, and 41.7%, respectively (34). These results indicate that the screening efficiency of the *pheS*m system typically exceeds 30%. In the current study, the efficiency of screening for the *ApglgB* mutant following the first step of recombination was nearly 100%, as the transformants were screened for the presence of the antibiotic resistance marker Erm^R^ via selection on erythromycin-containing medium (Fig. 2C). Subsequently, a marker-free cassette was introduced into the *ApglgB* mutant via natural transformation to achieve a second round of homologous recombination, and transformants were subjected to counterselection in the presence of 20 mM *p*-Cl-Phe. This process resulted in the successful generation of the marker-free *ApglgB::FtlpxE* strain with a screening efficiency of 58.57% ± 4.78% (Fig. 3D). Additionally, the two-step strategy based on the *AppheS*m system was employed for generating marker-free *ApcpsB* and *ApneuB* mutant strains with screening efficiencies of 70.11% ± 5.97% (Fig. 6I) and 63.87% ± 7.05% (Fig. 6J), respectively. These results confirm the feasibility and efficiency of employing *AppheS*m as a counterselectable marker in *Av. paragallinarum*. To the best of our knowledge, this is the first study to demonstrate the application of mutant alleles of *AppheS* as counterselectable markers for effective and efficient genetic engineering in *Av. paragallinarum*.

The *AppheS*m counterselectable marker system described herein not only facilitates seamless gene knock-out but also enables the direct insertion and expression of exogenous genes in *Av. paragallinarum*. As a proof of concept, the exogenous gene *FtlpxE* was seamlessly integrated into the genome of *Av. paragallinarum* using the *AppheS*m system. Following integration, the gene *FtlpxE* was effectively expressed, yielding a functional enzyme whose activity (the removal of a phosphate group from lipid A) was demonstrated herein. The *AppheS*m system, therefore, allows the excision of target genes and the insertion of specific exogenous genes into target loci without employing antibiotic resistance markers for screening following the second round of homologous recombination, thereby alleviating concerns over the emergence of antibiotic-resistant superbugs. The disaccharide backbone of lipid A is extensively preserved in the majority of bacterial species (38, 39) and is bonded with mono- or bi-phosphorylated cations at positions C-1 and C-4 (40). The toxicity of lipid A is determined by its structural characteristics, including the quantity and size of fatty acids, as well as the presence of phosphorylated polar functional groups such as phosphoethanolamine (PEtN) (41, 42). Considerable variations in the structure of lipid A are evident not only across different bacteria, but also within individual species. The lipid composition of gram-negative bacteria is frequently altered to enhance intracellular survival; an example is the structural modification of lipids via the incorporation of pEtN (43). Furthermore, these alterations aid the process of infection by allowing the bacteria to escape detection by the toll-like receptor 4, a key activator of the innate immune response (44). To the best of our knowledge, the current study is the first to analyze the composition of lipid A in *Av. paragallinarum* (Fig. 4). The analysis revealed the presence of two types of lipid A structures, one of which contains pEtN. The LpxE from *F. novicida* can dephosphorylate lipid A at the C-1 position (30). This activity was further confirmed by the partial dephosphorylation of lipid A observed in the mutant *ApglgB::FtlpxE* (Fig. 4B) but not the wild-type strain SC05. These findings provide evidence for the successful expression and functional activity of LpxE in the *Av. paragallinarum* strain SC05. Lipid A plays a crucial role in bacterial infections, and excessive activation of the immune system by this biomolecule can result in sepsis and fatal shock (45). The strain constructed herein harbors the exogenous gene *FtlpxE* integrated into the genome, which may potentially decrease the risk associated with such an activation and reduce the side effects of vaccines, although further experiments are warranted to confirm the same.

The present investigation additionally evaluated the phenotypic characteristics of the *ApcpsB* and *ApneuB* mutant strains. A previous study indicated that the *acbEDGHCB* and *ccbEF1F2CB* loci may contribute to CPS synthesis in *Av. paragallinarum* (46). Another study reported that the deletion of the genes *acbD* or *ccbF1* significantly impacts CPS production (33); however, the association between the mannose-1-phosphate guanylyltransferase CpsB and capsule formation remains to be elucidated. Due to the lack of appropriate antibody tools for CPS detection, we were unable to clearly assess the impact of *ApcpsB* on CPS biosynthesis. Nevertheless, the complement sensitivity results suggest that the loss of *ApcpsB* likely influences the synthesis of specific polysaccharides in *Av. paragallinarum*. Multiple studies have shown that the gene *neuB* encodes sialic acid synthase (47–50). One of three open reading frames from *Campylobacter jejuni* that exhibits highest similarity with NeuB sequences from *E. coli* has been discovered to play a role in the production of LOS (51). Furthermore, the genes *mnaA*, *neuB*, *neuA*, *wecA*, *wzt*, and *wzm* are known to be involved in the biosynthesis and translocation of the O-chain of lipopolysaccharides (LPS) in *Legionella pneumophila* (52). These investigations indicate a role for *neuB* in the synthesis of LPS or LOS. However, sialic acid is generally located on the outermost surface of the core polysaccharides in LOS (53), and its absence may have little effect on the size of LOS. This may explain the lack of obvious differences in the size of LOS obtained from the *ApneuB* mutant of *Av. paragallinarum* compared to that from the wild-type strain SC05 (Fig. 7B).

Serum resistance is a virulence determinant in several species of the family *Pasteurellaceae* (54–56). Herein, statistically significant differences (*P* < 0.001) were observed in the sensitivities of the wild-type, *ApcpsB*, and *ApneuB* mutants to both chicken and porcine sera (Fig. 7C and D). Interestingly, the survival rate of all the tested strains in the presence of chicken serum exceeded 100%, except for the *ApcpsB* mutant strain. This indicates that chicken serum fails to exert a lethal effect on *Av. paragallinarum*, highlighting the high pathogenicity of the bacterium, which renders the complement system of chicken ineffective in eliminating the pathogen. By contrast, the survival rate of the *ApcpsB* mutant strain in these settings was merely 86.54% ± 8.5%, suggesting that the expression of *ApcpsB* significantly contributes to the resistance of the pathogen against the host complement system. Furthermore, all the tested strains exhibited survival rates of below 100% in porcine serum; this observation suggests that the bactericidal potential of the mammalian complement system may be stronger than that of the avian ones, at least with respect to *Av. paragallinarum*. A possible reason is that the predominant immunoglobulin in poultry is IgY, which differs from mammalian IgG in that it cannot bind to the complement component C1q (57). Consequently, IgY is unable to initiate the classical complement pathway, which may explain the relatively lower antibacterial activity of avian serum. Moreover, since *Av. paragallinarum* occurs exclusively in poultry, it is plausible that this bacterium has evolved to withstand only a relatively weaker complement system.

The current study demonstrates the feasibility of employing the *pheS*m system in *Av. paragallinarum* and proposes a two-step strategy as an effective tool for gene editing (including gene knock-out and knock-in) in the bacterium. This study represents a significant advancement in demonstrating the potential application of *AppheS*m as a counterselectable marker in *Av. paragallinarum*. Furthermore, the method developed herein is crucial for facilitating genetic manipulation of this bacterium.

## MATERIALS AND METHODS

### Bacterial strains, plasmids, and culture conditions

The bacterial strains and plasmids used in this study are listed in Tables 1 and 2, respectively. *E. coli* DH5α was cultured in LB medium or LB agar at 37°C. Unless otherwise indicated, *Av. paragallinarum* strains were cultured at 37°C in the presence of 5% carbon dioxide (CO_2_) in LB medium or LB agar supplemented with 10% chicken serum and 0.01% NAD; *p*-Cl-Phe (20 mM) was added to the LB agar for counterselection. The following antibiotics were employed at the indicated concentrations for screening transformants: ampicillin trihydrate (100 µg/mL) and gentamicin (30 µg/mL) for *E. coli*, and erythromycin (5 µg/mL), and gentamicin (18 µg/mL) for *Av. paragallinarum*.

**TABLE 1:** Strains used in this study^*a*^

Strain	Relevant characteristic(s)	Source
DH5α	F⁻, φ80d/*lacZ*ΔM15, Δ(*lacZYA-argF*) U169 *recA*1 *endA*1 *hsdR*17	Laboratory collection
BL21 (DE3)	F⁻, *ompT hsdS_B_* (r_B_⁻, m_B_⁻) *gal dcm* (DE3)	Laboratory collection
SC05	*Av. paragallinarum* clinical isolate	Laboratory collection
JS01	SC05, containing pJSF01 with *AppheS*m gene, Gm^R^	This study
JS02	SC05, Δ*ApglgB*, *ApglgB* disrupted by *AppheS*m and Erm^R^	This study
JS03	SC05, Δ*ApcpsB*, *ApcpsB* disrupted by *AppheS*m and Erm^R^	This study
JS04	SC05, *ApneuB*, *ApneuB* disrupted by *AppheS*m and Erm^R^	This study
JS05	SC05, *ApglgB*::*FtlpxE*, *ApglgB* insertion by *FtlpxE*	This study
JS06	SC05, *ApcpsB* marker-free mutant	This study
JS07	SC05, *ApneuB* marker-free mutant	This study
JS08	JS06 containing pJSF08 with intact *ApcpsB* gene, Gm^R^	This study
JS09	JS07 containing pJSF09 with intact *ApneuB* gene, Gm^R^	This study
JS10	SC05, the *AppheS*m and Erm^R^ cassettes were inserted immediately downstream of the stop codon of the *ApompA* gene.	This study
JS11	SC05, insertion of a His-tag immediately upstream of the *ApompA* stop codon.	This study
JS12	pJSF12 in BL21 (DE3), Kan^R^	This study

^*a*^ Gm^R^: gentamicin resistance; Erm^R^: erythromycin resistance; Kan^R^: kanamycin resistance.

**TABLE 2:** Plasmids used in this study^*a*^

Plasmid	Relevant characteristic(s)	Source
pUC19	Expression vector, Amp^R^	Novagen
pUC19-pD70 ori	pD70 ori in pUC19	This study
pUC19-Erm	Erythromycin resistance gene in pUC19	This study
pSF118	A shuttle plasmid containing pD70 ori and ColE1 ori sequences, Gm^R^	This study
p34S-gm	Gm resistance cassette-carrying vector, Gm^R^	(58)
pET-28b(+)	Expression vector, Kan^R^	Novagen
pET-28b(+)-*AppheS*m	*AppheS*m gene in pET-28b(+), Kan^R^	This study
pACYCduet-1	Expression vector, Cm^R^	Novagen
pACYCdue-1-lpxE	*FtlpxE* gene in pACYCdue-1	This study
pJSF01	Shuttle plasmid containing *AppheSm* gene in pSF118, Gm^R^	This study
pJSF02	Counterselectable cassette containing Erm^R^, *AppheSm* gene, the upstream and downstream sequences of *ApglgB* in pUC19, Erm^R^, Amp^R^	This study
pJSF03	Counterselectable cassette containing Erm^R^, *AppheSm* gene, the upstream and downstream sequences of *ApcpsB* in pUC19, Erm^R^, Amp^R^	This study
pJSF04	Counterselectable cassette containing Erm^R^, *AppheSm* gene, the upstream and downstream sequences of *ApneuB* in pUC19, Erm^R^, Amp^R^	This study
pJSF05	Markerless cassette containing *FtlpxE* gene, the upstream and downstream sequences of *ApglgB* in pUC19, Amp^R^	This study
pJSF06	Markerless cassette containing the upstream and downstream sequences of *ApcpsB* in pUC19, Amp^R^	This study
pJSF07	Markerless cassette containing the upstream and downstream sequences of *ApneuB* in pUC19, Amp^R^	This study
pJSF08	Complementary plasmid containing *ApcpsB* gene in pSF118, Gm^R^	This study
pJSF09	Complementary plasmid containing *ApneuB* gene in pSF118, Gm^R^	This study
pJSF10	Counterselectable cassette containing *AppheS*m and ErmR between the upstream and downstream arms flanking the stop codon of *ApompA*, Erm^R^, Amp^R^	This study
pJSF11	Markerless cassette containing His-Tag sequence between the upstream and downstream arms flanking the stop codon of *ApompA* in pUC19; ErmR, AmpR	This study
pJSF12	*ApompA* gene with C-terminal His-Tag in pET-28a(+), Kan^R^	This study

^*a*^ Gm^R^: gentamicin resistance; Amp^R^: ampicillin resistance; Erm^R^: erythromycin resistance; Cm^R^: chloramphenicol resistance; Kan^R^: kanamycin resistance.

### Bioinformatics analysis

Multiple sequence alignment was performed using Clustal X version 2.0 (59) and ESPript 3.0 (60). The amino acid sequences encoded by *pheS* from *E. coli* (NP416229.1), *R. anatipestifer* (WP004919587.1), *P. multocida* (WP005756547.1), *G. parasuis* (WP160405289.1), and *Av. paragallinarum* (WP035684599.1) were compared and analyzed.

### Evaluation of sensitivity of *Av. paragallinarum* strains to *p*-Cl-Phe

To investigate the sensitivity of *Av. paragallinarum* to *p*-Cl-Phe, the first step is the construction of the shuttle vector. The primers used in this study are listed in Table S1. Based on a previous investigation of replicons from bacteria of the family *Pasteurellaceae* (61), the wild-type replicon pD70 ori (accession number: DQ125466.1) was synthesized and introduced into the plasmid pUC19 (62) via seamless cloning (ClonExpress MultiS One Step Cloning Kit, Vazyme, Nanjing, China), thereby generating pUC19-pD70. The fragment containing pD70 ori was amplified by PCR using the primers P1 and P2, and the *E. coli* replicons and gentamicin resistance marker were amplified from p34S-Gm (58) using the primers P3 and P4; a new shuttle vector, pSF118, was generated by the seamless ligation of these two fragments. Simultaneously, the gene *AppheS* harboring a point mutation (A294G) was synthesized and cloned into the multiple cloning site of pET-28b(+) to generate pET-28b(+)-*AppheS*m. Subsequently, the *AppheS*m gene fragment of approximately 1,100 bp from pET-28b(+)-*AppheS*m was amplified by PCR using primers P5 and P6 (which contained target sites for the restriction enzymes *EcoRI* and *XhoI*, respectively). The amplified *AppheS*m fragment was then ligated with the *EcoRI*- and *XhoI*-digested pSF118 vector to obtain the shuttle plasmid pJSF01, which will be used to assess the sensitivity of *Av. paragallinarum* to *p*-Cl-Phe.

The wild-type *Av. paragallinarum* strain SC05 was transformed with the plasmid pJSF01 via electroporation. The strain was first grown overnight in LB broth supplemented with 10% chicken serum and 0.01% NAD at 37°C. The overnight culture was then transferred into fresh LB broth with the same supplements and incubated with shaking until the optical density at 600 nm (OD_₆₀₀_) reached 0.8. The cells were harvested by centrifugation and washed four times with 10% glycerol under cold conditions to prepare electrocompetent cells. Subsequently, 1  µg of plasmid DNA was mixed with the competent cells, transferred into a 1 mm electroporation cuvette, and subjected to electroporation using a Gene Pulser Xcell Electroporation System (Bio-Rad, California, USA) at 1800 V/cm, 50 µF capacitance, and 200 Ω pulse resistance. Following transformation, strain recovery was accomplished via incubation in LB broth supplemented with 10% chicken serum and 0.01% NAD for 3 h at 37°C with shaking at 200 rpm. The transformants were then plated on LB agar containing the same supplements and 18 µg/mL gentamicin, followed by incubation for 24–48 h at 37 °C to screen for the recombinant strain JS01. Sixteen-hour cultures of the strains SC05 and JS01 were diluted 100-fold in fresh LB medium, incubated under rotary conditions (200 rpm) at 37°C until an optical density at 600 nm (OD_600_) of 0.6 was attained, and then subjected to counterselection on LB plates containing *p*-Cl-Phe (5, 10, 15, and 20 mM) for 24–48 h at 37°C. At least three independent replicates were employed for each strain.

### Generation and identification of marker-free *ApglgB* mutant strains

The overall strategy for marker-free strains construction is shown in Fig. 9. First, we designed primers to amplify the flanking fragments of the target gene and the marker genes, including the upstream fragment, downstream fragment, ErmR, and *AppheS*m. Subsequently, the four fragments were assembled using overlap PCR and seamlessly cloned into the pUC19 vector. In the second step, the knock-out cassette containing *AppheS*m was introduced into *Av. paragallinarum* via natural transformation, followed by homologous recombination to obtain mutant strains with the genome-integrated knock-out cassette. In the third step, primers were designed to amplify the flanking fragments of the target gene. Similarly, the upstream and downstream fragments were joined using overlap PCR and cloned into the pUC19 vector. The extracted plasmid was then introduced into the previously obtained mutant strain via natural transformation, ultimately generating a marker-free mutant strain. It is worth noting that the primers for mutant strain verification were designed outside the primers used for amplifying the upstream and downstream fragments. This allows for the determination of whether genomic editing has occurred at the target locus.

The first step is the design and construction of the knock-out cassette, using the construction of the *ApglgB* knock-out cassette as an example. The counterselectable marker cassette was constructed by overlap extension PCR using DNA from the wild-type strain SC05 as a template and the primer pairs P7/P8 (for amplifying a 928 bp DNA fragment encompassing the upstream region of the gene *ApglgB*) and P9/P10 (for amplifying the 936 bp region downstream of the *ApglgB* locus). The *AppheS*m gene fragment (1,030 bp) was amplified by PCR from pET-28b(+)-*AppheS*m using the primer pair P11/P12, and the Erm^R^ cassette (857 bp) was amplified from the plasmid pUC19-Erm^R^ using the primer pair P13/P14. The resulting four fragments were connected by overlap extension PCR using the primers P7 and P10 to generate the cassette *ApglgB::AppheS*m. The plasmid pUC19 was amplified with the primer pair P15/P16, and the *ApglgB::AppheS*m cassette was seamlessly ligated into pUC19 to generate the plasmid pJSF02. It is worth noting that the core DNA uptake signal sequence (USS) 5′-ACCGCACTT-3′ (19) was retained in each upstream region, which was introduced through the forward primers.

The *ApglgB* marker-free knock-in strain was constructed in this study using a two-step strategy based on natural transformation (19). The first step involved the generation of the *ApglgB* insertion mutant containing an *ApglgB::AppheS*m knock-out cassette at the *ApglgB* locus on the genome. The strain SC05 was cultured for 16 h, diluted 100-fold in fresh LB broth, and incubated at 37°C under rotary conditions (200 rpm) until an OD_600_ of 0.6 was attained. The culture was thoroughly mixed with the plasmid pJSF02 and incubated in LB broth containing 10% chicken serum and 0.01% NAD at 37°C for 2 h. The resulting transformants (strain JS02) were screened on LB agar plates supplemented with 5 µg/mL erythromycin and incubated in the presence of 5% CO_2_ at 37°C for 24–48 h. Single colonies on the plates were subjected to PCR analysis using the primers P31 and P32 to identify the successful insertion of the *ApglgB::AppheS*m knock-out cassette in the genomic DNA of strain SC05, with amplicon sizes of 4,411 and 2,536 bp corresponding to the strains JS02 and SC05, respectively. The PCR products of expected sizes were sequenced for verifying the appropriate insertion of the gene *AppheS*m.

A similar strategy was employed for the second step, which involved the replacement of the *ApglgB::AppheS*m knock-out cassette in the strain JS02 with the *ApglgB::FtlpxE*-marker-free cassette. For the construction of the marker-free knock-in cassette, the gene *FtlpxE* from *Francisella tularensis* subsp. *novicida* (accession number: CP000439.1) was synthesized and cloned into the plasmid pACYCDuet-1 (Merck Millipore, USA) to yield the plasmid pACYCDuet-1-*FtlpxE*; the *FtlpxE* gene fragment was then amplified by PCR using the primers P29/P30, and the *ApglgB::FtlpxE*-marker-free cassette, containing the gene *FtlpxE* flanked by homology arms of the *ApglgB* gene, was constructed by overlap extension PCR using the primers P7/P10. This cassette contained the above-mentioned USS sequence (which was introduced via the forward primer) and was cloned into the plasmid pUC19 to generate pJSF05. The plasmid pJSF05 was introduced into the strain JS02 via natural transformation. The plasmid pJSF05 was mixed with the strain JS02 and incubated in LB broth containing 10% chicken serum and 0.01% NAD at 37°C for 4 h. The cells were then spread onto LB agar plates containing 20 mM *p*-Cl-Phe to allow the counterselection of the marker-free *ApglgB* mutant and incubated at 37°C in the presence of 5% CO_2_ for 24–48 h. The transformants were identified using the primers P31 and P32. A successful replacement of the *ApglgB::AppheS*m knock-out cassette with *ApglgB::FtlpxE*-marker-free is expected to yield an amplicon of 3,208 bp, compared with 4,411 bp obtained with the strain JS02. The marker-free knock-in strain at the *ApglgB* locus was successfully generated, resulting in JS05.

### Generation and identification of marker-free *ApcpsB* and *ApneuB* mutant strains

The insertion knock-out cassettes *ApcpsB::AppheS*m and *ApneuB::AppheS*m were similarly generated, but using different primers. Briefly, the upstream (592 bp) and downstream (503 bp) regions of the gene *ApcpsB* were amplified from the genomic DNA of the strain SC05 using the primers P17, P18, P19, and P20. Subsequently, these two fragments were merged with the *AppheS*m and Erm^R^ fragments to yield the *ApcpsB::AppheS*m cassette by overlap extension PCR using the primers P17/P20. The upstream (552 bp) and downstream (436 bp) regions of the gene *ApneuB* were amplified from the genomic DNA of strain SC05 using the primers P21, P22, P23, and P24; these fragments were subsequently merged with the *AppheS*m and Erm^R^ fragments to yield the *ApneuB::AppheS*m cassette by overlap extension PCR using the primers P21/P24. These cassettes were subsequently cloned into the plasmid pUC19 by seamless ligation, as detailed above. The plasmids containing the *ApcpsB::AppheS*m and *ApneuB::AppheS*m cassettes are referred to as pJSF03 and pJSF04, respectively.

Marker-free cassettes can be designed to achieve knock-out of endogenous genes as well as knock-in of exogenous genes and are constructed using overlap extension PCR. The *ApcpsB*-marker-free and *ApneuB*-marker-free cassettes were designed to achieve marker-free knock-out of the genes *ApcpsB* and *ApneuB*, respectively. The primers P17/P25 and P26/P20 were used to amplify the upstream and downstream regions, respectively, of the gene *ApcpsB* from the genomic DNA of the strain SCO5 and incorporated into the *ApcpsB-*marker-free cassette by overlap extension PCR using the primers P17/P20. The *ApneuB*-marker-free cassette was similarly generated using a different set of primers; the upstream and downstream regions of *ApneuB* were amplified by PCR using the primers P21/P27 and P28/P24, respectively, and incorporated into the *ApneuB*-marker-free cassette by overlap extension PCR using the primers P21/P24. All these cassettes contained the abovementioned USS sequence (which was introduced via the forward primer) and were cloned into the plasmid pUC19. The plasmids containing the *ApcpsB*-marker-free and *ApneuB*-marker-free cassettes have been referred to as pJSF06 and pJSF07, respectively.

The construction of *ApcpsB* and *ApneuB* marker-free mutant strains followed a similar approach to that used for the *ApglgB* mutant strain. Specifically, pJS03 was used for natural transformation to generate the *ApcpsB* insertion mutant strain JS03, while pJS06 was used for transformation to obtain the *ApcpsB* marker-free mutant strain JS06. For *ApneuB*, pJS04 was used to generate *ApcpsB* insertion mutant strain JS04, while pJS07 was used to obtain the *ApneuB* marker-free mutant strain JS07. The strains harboring genomic deletion of the genes *ApcpsB* and *ApneuB* were constructed as detailed above for *ApglgB*, except that different primers were employed for PCR-based screening. The primers P33/P34 were used to amplify the *ApcpsB* locus, yielding PCR amplicons of sizes 3,908, 1,308, and 1,849 bp, corresponding to the strains JS03, JS06, and SC05 (wild-type strain), respectively. The primers P35/P36 were employed for amplifying the *ApneuB* locus, yielding PCR amplicons of sizes 3,745, 1,134, and 1,645 bp, corresponding to the strains JS04, JS07, and SC05 (wild-type strain), respectively.

### Construction and identification of *ApcpsB* and *ApneuB* mutants complemented with the corresponding genes

To generate the *ApcpsB* complement strain, the complete *ApcpsB* gene (1,089 bp) was amplified using the primer pair P37/P38, while the shuttle plasmid pSF118 was amplified using the primer pair P39/P40. These two fragments were then seamlessly ligated to generate the plasmid pJSF08. The plasmid pJSF08 was introduced into the strain JS06 via electroporation. Following recovery, the culture was evenly distributed on LB agar plates supplemented with gentamicin (18 µg/mL). The plates were then incubated at 37°C in the presence of 5% CO_2_ for 24–48 h. Subsequently, the positive transformants (yielding an amplicon of approximately 1,000 bp) were detected by colony PCR using the primer pair P41/P42 and termed as the *ApcpsB-*complemented mutant strain JS08. The *ApneuB-*complemented mutant strain was similarly generated, except that different primers were employed; the primers P43 and P44 were employed for amplifying the complete *ApneuB* gene (1,038 bp), which was then cloned into plasmid pSF118 to construct the plasmid pJSF09. The plasmid pJSF09 was then introduced into the strain JS07 as detailed above, and the transformants (yielding an amplicon of size about 1,000 bp and termed the *ApneuB-*complemented mutant strain JS09) were identified by PCR using the primers P41 and P42.

### Construction and characterization of *Av. paragallinarum* with a His-tag introduced *ApompA*

To construct a strain with a His-tagged OmpA, the upstream (539 bp) and downstream (387 bp) regions of the gene *ApompA* were amplified from the wild-type strain SC05 using the primers P45, P46, P47, and P48. Subsequently, these two fragments were connected with the *AppheS*m and Erm^R^ fragments to yield the *ApompA-*His-tagged::*AppheS*m cassette by overlap PCR using the primers P45/P48. This cassette was then cloned to pUC19, as mentioned above, to generate pJSF10. Subsequently, this plasmid was used to construct the *ApompA* C-terminal insertion strain, JS10. The upstream and downstream fragments of marker-free His-tagged *ApompA* cassette were amplified using primers P45/P49 and P47/P48 and then connected with primers P45 and P48. The cassette was seamlessly cloned into the pUC19 vector as mentioned above to generate pJSF11. Next, this plasmid was used for the construction of the marker-free His-tagged *ApompA* strain, resulting in strain JS11. The strain JS11 was confirmed by PCR, sequencing, and Western blot.

### Isolation and identification of lipid A

Lipid A was isolated as detailed previously (31, 63), but with certain modifications to the procedure. The strain was cultured in a conical flask containing 100 mL of LB medium and incubated for 16 h at 37°C under rotary conditions (200 rpm). The culture was then centrifuged (8,000 × *g*, 10 min) to harvest the cells, followed by the addition of lysis buffer (30 mL; water, chloroform, and methanol in a ratio of 0.8:1:2) and cell lysis via two rounds of crushing in a cell crusher for a period of 10 min each. The lysed cells were then subjected to centrifugation (8,000 × *g*, 10 min), followed by the removal of the supernatant. Subsequently, a solution of citric acid (30 mL, 0.1 M, pH 2.9) was added and mixed with the cell pellet. The mixture was then treated in a boiling water bath (100°C) for 90 min to achieve complete hydrolysis of LOS and the release of lipid A. The precipitate obtained following centrifugation was again collected, dried, and stored at –20°C. MALDI-TOF MS analysis was carried out using the UltrafleXtreme instrument (Bruker Daltonik GmbH, Germany) in negative-ion mode. Samples were mixed with 2,5-dihydroxybenzoic acid matrix solution (10 mg/mL) and were analyzed using MS. The mass spectra corresponding to lipid A (m/z range of 900–2,500) were documented.

### Extraction and analysis of CPS and LOS

The extraction of CPS was carried out as detailed previously (46), with a few modifications. The bacteria cultured on LB agar plates were resuspended in phosphate-buffered saline (PBS), harvested via centrifugation (10,000 × *g*, 10 min), and subsequently rinsed again with PBS. The cells were then resuspended in 1 mL of extraction buffer (50 mM Tris-HCl and 5 mM ethylenediaminetetraacetic acid, pH 7.3) and incubated for 30 min at 37°C. Hexadecyltrimethylammonium bromide was added at a final concentration of 0.5% (wt/vol), and the cells were incubated for 16 h at room temperature. The mixture was then subjected to centrifugation (10,000 × *g*, 10 min), and the resulting precipitate was dissolved in 1 M sodium chloride (0.2 mL). The solution was then mixed with Trizol reagent (1 mL) and chloroform/isoamyl alcohol (24:1, 0.2 mL). The liquid obtained was collected, mixed with four times its volume of ethanol, and incubated at −70°C for 1 h to precipitate the CPS. Following centrifugation (10,000 × *g*, 10 min), the solid precipitate containing the CPS was dissolved in 0.5 mL of buffer (10 mM Tris-HCl, 50 mM potassium chloride, and 1.5 mM magnesium chloride, pH 8.3). The sample was then treated with DNase I (Takara, Japan) and RNase A (Sigma-Aldrich, USA) at 37°C for 1 h to remove any residual DNA and RNA. The CPS samples were analyzed using gradient (4%–20%) SDS–PAGE, followed by silver staining. LOS extraction was carried out as previously described (64); the LOS samples were separated on a 15% SDS–PAGE gel and visualized by silver staining.

### Serum bactericidal assay

Chicken and porcine sera were obtained from the Laboratory Animal Center of South China, China. The sera were subjected to filter sterilization using 0.22 μm filters, and aliquots were stored at −80°C. An aliquot of the serum samples was inactivated by heat treatment at 56°C for 30 min. The serum bactericidal assay was carried out with porcine and chicken sera as per a previously outlined protocol (65). To achieve a final serum concentration of 90%, 0.1 mL of the bacterial culture, grown to logarithmic phase (approximately 1 × 10^8^ CFU/mL), was added to 0.9 mL of fresh or heat-treated chicken sera. The reaction mixtures were incubated under moderate shaking conditions at 37°C for 1 h. The mixtures were then subjected to 10-fold serial dilutions, and a 0.1 mL aliquot from each dilution was plated in triplicate on LB agar plates supplemented with 10% chicken serum and 0.01% NAD. The plates were then incubated for 24–48 h at 37°C in the presence of 5% CO_2_, and the colonies were counted. A similar experimental protocol was followed for porcine serum, except that a serum concentration of 30% was employed. The survival ratio was calculated by comparing the number of colonies obtained upon treatment with fresh serum to those with heat-treated serum. Each strain of *Av. paragallinarum* was evaluated in three independent experiments.

### Southern blot analysis

To confirm genes marker-free knock-outs by Southern blotting, 5 μg of gDNA from the *ApglgB* mutant was digested with KpnI, gDNA from the *ApneuB* mutant or *FtlpxE* integrated strain was digested with NheI, and gDNA from *ApcpsB* mutant was treated with EcoRI for 16 h at 37°C. Products of restriction enzyme digestion were resolved by electrophoresis in a 0.8% agarose gel at 30 V for 16 h, and the resulting fragments were transferred to a nylon membrane via capillary blotting. The probe fragments were amplified and cloned into the pUC19 vector. The resulting recombinant plasmids were then used as templates for probe amplification with primers P50/P51 for *ApglgB*, P52/P53 for *ApneuB*, and P54/P55 for *ApcpsB*. The prepared probes were labeled using the DIG-High Prime DNA Labeling and Detection Starter Kit I (Roche, Germany), and hybridization was performed according to the manufacturer’s instructions.

### Expression and purification of ApOmpA

The *ApompA* gene was amplified from the genome of wild-type strain SC05 using primers P56 and P57 and seamlessly cloned into the HindIII-digested pET-28(a) vector to generate the expression plasmid pJSF12. This plasmid was transformed into *E. coli* BL21(DE3) to obtain the ApOmpA expression strain JS12. Cultures were grown at 37 °C to an OD_₆₀₀_ of approximately 0.6, induced with 0.5 mM IPTG, and incubated for 4 h. Cells were harvested by centrifugation, resuspended in lysis buffer (10 mM imidazole, 300 mM NaCl, 50 mM sodium phosphate, pH 8.0), and disrupted by sonication. Inclusion bodies were recovered by centrifugation and washed with lysis buffer containing 1% Triton X-100 to remove contaminants. The pellet was solubilized in lysis buffer with 8 M urea, and the protein was purified by Ni–NTA affinity chromatography under denaturing conditions. Bound proteins were washed and eluted with buffers containing either 10 mM or 300 mM imidazole, 300 mM NaCl, 50 mM sodium phosphate (pH 8.0), and 8 M urea.

### Western blot analysis

Cultures of the wild-type (WT) strain and the OmpA-His-tag recombinant strain were subjected to ultrasonication to disrupt the cells, followed by centrifugation to collect the pellets. The resulting pellets were resuspended in phosphate-buffered saline (PBS) and separated by 12% SDS–PAGE. Subsequently, immunoblotting was performed using a monoclonal anti-His-tag antibody (GenScript, China), and protein bands were visualized with the Immobilon Western chemiluminescent HRP substrate (Millipore, USA).

### Transcriptomic analysis

Total RNA was isolated using the Trizol Reagent (Invitrogen Life Technologies, USA). Quality and integrity were determined using a NanoDrop spectrophotometer (Thermo Scientific, USA) and a Bioanalyzer 2100 system (Agilent). Zymo-Seq RiboFree Total RNA Library Kit (Zymo Research, USA) was used to remove rRNA from total RNA. Random oligonucleotides and SuperScript III were used to synthesize the first-strand cDNA. Second-strand cDNA synthesis was subsequently performed using DNA Polymerase I and RNase H. The remaining overhangs were converted into blunt ends via exonuclease/polymerase activities, and the enzymes were removed. After adenylation of the 3′ ends of the DNA fragments, Illumina PE adapter oligonucleotides were ligated to prepare for hybridization. To select cDNA fragments of the preferred 400–500 bp in length, the library fragments were purified using the AMPure XP system (Beckman Coulter, USA). DNA fragments with ligated adaptor molecules on both ends were selectively enriched using Illumina PCR Primer Cocktail in a 15-cycle PCR reaction. Products were purified (AMPure XP system) and quantified using the Agilent high sensitivity DNA assay on a Bioanalyzer 2100 system (Agilent, USA). The sequencing library was then sequenced on the NovaSeq 6000 platform (Illumina, USA) by Shanghai Personal Biotechnology Co., Ltd.

The quality information of raw data in FASTQ format was calculated, and then the raw data were filtered using Cutadapt (v1.15) software. Clean data were obtained by removing reads containing adapter, reads containing poly-N, and low-quality reads. All subsequent analyses were based on high-quality clean data. The reference genome and gene annotation files were downloaded from NCBI website (accession No: CP113955). The reference genome index was built using Bowtie2 (v2.2.6), and the filtered reads were mapped to the reference genome using Bowtie2 (http://bowtie-bio.sourceforge.net/index.shtml). The gene read count value was counted using HTSeq (v0.9.1) as the original expression level of the gene. In order to make the gene expression levels of different genes and different samples comparable, FPKM (fragments per kilobase of exon per million fragments mapped) is used to normalize the expression. DESeq (v1.30.0) was conducted to analyze the differential expressed mRNA. Transcripts with |log2FoldChange|>1 and *P*-value < 0.05 were considered as differentially expressed mRNA. Volcano maps and MA maps of differentially expressed mRNAs were generated using the R language ggplot2 software package. The complete transcriptome analysis (Data Set S1) is provided in the supplementary materials.

### Scanning electron microscopy

Bacterial pellets after serum treatment were harvested by centrifugation, with each pellet reaching at least the size of a rice grain. Remove the culture medium, then wash the bacteria with PBS, and remove the PBS. Add the fixative to the tube and let the precipitation re-suspend in the fixative. For post-fixation, the bacterial pellets were washed three times with 0.1 M PB (pH 7.4) for 15 min each, followed by incubation in 1% OsO₄ in 0.1 M PB (pH 7.4) for 1–2 h at room temperature. The samples were then washed again three times in 0.1 M PB (pH 7.4) for 15 min each. Dehydration was performed sequentially in 30%, 50%, 70%, 80%, 90%, and 95% ethanol for 15 min each, followed by two changes of 100% ethanol for 15 min, and finally transferred to isoamyl acetate for 15 min. Samples were dropped onto coverslips and dried using a critical point dryer. The dried specimens were mounted on metallic stubs with carbon adhesive and sputter-coated with gold for 30 s. Prepared samples were then examined and imaged using a scanning electron microscope.

### Transmission electron microscopy

Bacterial pellets after serum treatment were collected by centrifugation, ensuring that each pellet reached at least the size of a mung bean. The pellets were resuspended in TEM fixative and fixed at 4°C for preservation. The fixed samples were centrifuged, the supernatant was removed, and the pellets were washed in 0.1 M phosphate buffer (PB, pH 7.4) for 3 min, repeating this three times. The 1% agarose solution was prepared in advance and cooled before use; the pellets were then suspended in the agarose prior to solidification and embedded within the agarose blocks. For post-fixation, the agarose blocks were incubated in 1% OsO₄ in 0.1 M PB (pH 7.4) for 2 h at room temperature in the dark, followed by three washes in 0.1 M PB for 15 min each. Dehydration was performed at room temperature through a graded ethanol series of 50%, 70%, 80%, and 95% ethanol for 20 min each, followed by two changes of 100% ethanol for 20 min and two changes of acetone for 15 min.

Resin infiltration was carried out sequentially in acetone:EMBed-812 mixtures of 1:1 for 2–4 h at 37°C, 1:2 overnight at 37°C, and then in pure EMBed-812 for 5–8 h at 37°C. Samples were embedded in fresh EMBed-812 in molds and kept at 37°C overnight. Polymerization was performed at 60°C for more than 48 h, after which the resin blocks were removed from the molds and stored at room temperature. Ultrathin sections (60–80 nm) were cut using an ultramicrotome and collected onto 150-mesh copper grids coated with formvar film. Sections were stained with 2% uranyl acetate in saturated alcohol for 8 min in the dark, rinsed in 70% ethanol and ultrapure water, and then stained with 2.6% lead citrate for 8 min while avoiding CO₂ exposure, followed by ultrapure water washes. After being dried on filter paper, the copper grids were placed on the grid board and left overnight to dry at room temperature. The copper grids were observed under a TEM, and images were taken.

### Statistical analysis

Each experiment was conducted at least three times, and the standard deviation was calculated using GraphPad Prism 9 (San Diego, CA, USA). Student’s *t*-test was employed for assessing the significance of the observed variations. *P*-values of < 0.05 were regarded as statistically significant.

## Data Availability

The authors confirm that the data for this study are available in the article and its supplementary material. Plasmid sequences based on *AppheS*m screening markers are listed in Table S2 in the supplemental material.

## SUPPLEMENTAL MATERIAL

Data Set S1 aem.02398-25-s0001.xls Transcriptome data.

Supplemental material aem.02398-25-s0002.docx Tables S1 and S2; Fig. S1.

ASM does not own the copyrights to Supplemental Material that may be linked to, or accessed through, an article. The authors have granted ASM a non-exclusive, world-wide license to publish the Supplemental Material files. Please contact the corresponding author directly for reuse.
