# Supplementary material for: Development of a counterselection system for efficient marker-free genetic manipulation in Avibacterium paragallinarum
Source: Appl Environ Microbiol. 2026 Mar 30;92(4):e02398-25. doi: 10.1128/aem.02398-25 (PMC13101520; doi:10.1128/aem.02398-25)

**Development of a counterselection system for efficient marker-free genetic manipulation in *Avibacterium paragallinarum***

**Authors:** Juan Sun^a^, Ling Chen^a^, Ge He^a^, Yizhen Lin^a^, Jialian Hu^a^, Ning Dai^a^, Xuewei Cao^a^, Yifeng Huang^a^, Yu Han^a^*, Saixiang Feng^a,b,c,d,e^*

^a^College of Veterinary Medicine, South China Agricultural University, Guangzhou, China.

^b^Key Laboratory of Zoonosis Prevention and Control of Guangdong Province, China.

^c^Key Laboratory of Zoonosis of Ministry of Agriculture and Rural Affairs, Guangzhou, China.

^d^Key Laboratory of Veterinary Vaccine Innovation of the Ministry of Agriculture and Rural Affairs, Guangzhou, China.

^e^National and Regional Joint Engineering Laboratory for Medicament of Zoonosis Prevention and Control, Guangzhou, China.

***Correspondence:**

Saixiang Feng: Tel/Fax, +86-20-85280718; E-mail: fengsx@scau.edu.cn

Yu Han: Tel/Fax, +86-20-85280718; E-mail: hanyuscau@126.com

**SUPPLEMENTARY FIGURE CAPTIONS**

**Figure S1.** SDS-PAGE analysis of LOS from complement-treated *cpsB* and *neuB* mutant strains. Lanes: M, marker; 1, Wild-type strain; 2, LOS from *cpsB* mutant; 3, LOS from *neuB* mutant.

**Supplementary Table**

**Table S1.** Primers used in this study.

| **Primers** | **Primer sequences (5′-3′)** |  |
| --- | --- | --- |
| P1 (pD70ori-F) | GATCCCTTTTTCTGTAATCTGTTTCGTG |  |
| P2 (pD70ori-R) | CTCGAGGCATAAGCGAATTCGATCATAGGCTCAATTCTCGC |  |
| P3 (p34SGm-F) | GAATTCGCTTATGCCTCGAGTTCGCTATTACGCCAGCTG |  |
| P4 (p34SGm-R) | CACGAAACAGATTACAGAAAAAGGGATCAGCCTGAATGGCGAATGGCG |  |
| P5 (*phe*S*m-*F) | GAATTCaaacgtgattttttaaggcgttagt |  |
| P6 (*phe*S*m-*R) | CTCGAGttatttaaattgttttaagaaacgcag |  |
| P7 (*glgB*-up-mut-F) | GCCTGCAGGTCGACTCTAGAGGtgcaccgcactttGTATTATTGCCTGATGCCAA |  |
| P8 (*glgB*-up-mut-R) | CTCAGTGATGCTAGGACGTCGGTATCACTTGGGAAATGCC |  |
| P9 (*glgB*-down-mut-F) | CTGAACTTCGTCACTCATCGGGCTAGAACGCTTTGGTTTA |  |
| P10 (*glgB*-down-mut-R) | GAATTCGAGCTCGGTACCCCGATGGCTTCACTCACCACC |  |
| P11 (*pheS*m-JT-F) | GACGTCCTAGCATCACTGAGAAACGTGATTTTTTAAGGCGTTAG |  |
| P12 (*pheS*m-JT-R) | GTACTCACGACGCTCAGTCTttatttaaattgttttaagaaacgcagatcg |  |
| P13 (*ErmR*-JT-F) | AGACTGAGCGTCGTGAGTACATGAACGAGAAAAATATAAAACACAGTCAAAAC |  |
| P14 (*ErmR*-JT-R) | CGATGAGTGACGAAGTTCAGCACACAGGAAACAGCTATG |  |
| P15 (pUC19-F) | GGGTACCGAGCTCGAATTC |  |
| P16 (pUC19-R) | CCTCTAGAGTCGACCTGCAGGC |  |
| P17 (*cpsB*-up-mut-F) | GCCTGCAGGTCGACTCTAGAGGtgcaccgcactttatggatagaatggaacattc |  |
| P18 (*cpsB*-up-mut-R) | CTCAGTGATGCTAGGAgtcttgctcgttcaatatttgg |  |
| P19 (*cpsB*-down-mut-F) | CTGAACTTCGTCACTCATCGctggttgaaagcaattcgtc |  |
| P20 (*cpsB*-down-mut-R) | GAATTCGAGCTCGGTACCCttattttgtattattctc |  |
| P21 (*neuB*-up-mut-F) | GCCTGCAGGTCGACTCTAGAGGtgcaccgcactttgatagggtgtaatcataacggtgatc |  |
| P22 (*neuB*-up-mut-R) | CTCAGTGATGCTAGGACGTCgtaggatattcggtattacagtgc |  |
| P23 (*neuB*-down-mut-F) | CTGAACTTCGTCACTCATCGgaagatgtaaatctaaattcaatcaatgc |  |
| P24 (*neuB*-down-mut-R) | GAATTCGAGCTCGGTACCCttagtcttggttaggaaatctactatctatg |  |
| P25 (*cpsB*-up-mf-R) | GTACTCACGACGCTCAGTCTTTGCTGATGAATCAAGGTGC |  |
| P26 (*cpsB*-down-mf-F) | AGACTGAGCGTCGTGAGTACGAGTCTCAATCTATCGATTATGCCG |  |
| P27 (*neuB*-up-mf-R) | GTACTCACGACGCTCAGTCTgggtcatagcaagttgtg |  |
| P28 (*neuB*-down-mf-F) | AGACTGAGCGTCGTGAGTACcaagcttcaatcactcc |  |
| P29 (*lpxE*-F） | GACGTCCTAGCATCACTGAGtagattagagtaatcaagtcaatagtttctaata |  |
| P30 (*lpxE*-R） | CGATGAGTGACGAAGTTCAGctaaataatctctctatttctcatccaata |  |
| P31 (*glgB*-test-F) | GCCGAAAGCCAAACCAAGCTTATC |  |
| P32 (*glgB*-test-R) | CGCCTTGATGACTTCCCGTC |  |
| P33 (*cpsB*-test-F) | cctatagcatcaacatctgc |  |
| P34 (*cpsB*-test-R) | gccagcaacaggccttgttg |  |
| P35 (*neuB*-test-F) | gtttgtgggtaagtatgcca |  |
| P36 (*neuB*-test-R) | ccttaatggtgatgtcggttg |  |
| P37 (*cpsB*-F) | ATGTGCTGCAAGGCGATTAAGgaatggatagaatggaacattcaatcactc |  |
| P38 (*cpsB*-R) | TGACTGGGAAAACCCTGGCGcacagtttattttgtattattctcattca |  |
| P39 (pSF118-F) | CTTAATCGCCTTGCAGCACAT |  |
| P40 (pSF118-R) | CGCCAGGGTTTTCCCAGTCA |  |
| P41 (pSF118-test-F) | CTTCGCTATTACGCCAGCTG |  |
| P42 (pSF118-test-R) | CCTAAATTCACTGGCCGTCG |  |
| P43 (*neuB*-F) | ATGTGCTGCAAGGCGATTAAGatgacaaaagtatttattactgctgagata |  |
| P44 (*neuB*-R) | TGACTGGGAAAACCCTGGCGcgttagtcttggttaggaaatctactatc |  |
| P45 (*ompA*-up-F) | GCCTGCAGGTCGACTCTAGAGGTGCACCGCACTTTcagagcttgcattacgtgttg |  |
| P46 (*ompA*-up-R) | CTCAGTGATGCTAGGACGTCctactcgttacctttaactg |  |
| P47 (*ompA*-down-F) | CTGAACTTCGTCACTCATCGttttttaacacgttaattcg |  |
| P48 (ompA-down-R) | GAATTCGAGCTCGGTACCCgaattgtgcgagctgctcttg |  |
| P49 (ompAhis-up-R) | CTCAGTGATGCTAGGACGTCctaATGATGATGATGATGATGctcgttacctttaactgaaatttc |  |
| P50 (glgB-probe-F) | ATTGCGGAAGAATCCACTTCT |  |
| P51 (glgB-probe-R) | ATCCAACTCATATAAAGCGGGGT |  |
| P52 (neuB-probe-F) | TGTTTCCGTTTCCTCTGCTCA |  |
| P53 (neuB-probe-R) | TGTCATTCGTTCCCAGCATTATAATA |  |
| P54 (cpsB-probe-F) | TGCTATAGGGACATTGCGGT |  |
| P55 (cpsB-probe-R) | CATCCCCTACATTCACGGCA |  |
| P56 (ompA-F for pET) | CTTTAAGAAGGAGATATACCatgaaaaaaactgcaatcgc |  |
| P57 (ompA-R for pET) | TTGTTAGCAGCCGGATCTCAttaATGATGATGATGATGATGgttaaaatttaccacag |  |

**Table S2.** Plasmid sequences in this study.

| **Plasmids** | **Plasmid sequences (5′-3′)** |
| --- | --- |
|  |  |
| pJSF01 (*AppheS*m) | CATATGGATCCCTTTTTCTGTAATCTGTTTCGTGCGTTCTTTGCTAAGATACAGACCCTAGACAAGTCATATCTTAGCAAAGGGTAGCTAGTAATGCAAGAGATTGCGAAGCGTCCCTACTACCAAAAAACCATTCAACGACGTAAACAGACAAACGCAAACCTTAAATTAGACGGTCTTCAGCTCGGACTTCGGAAGAATAAACAGGCGTAGAAGTGATAACGTTCTTAATACGAAAATTAAGCTCTGTCTCCGTTTCGTGCTACGGTTAGAAAGGCGAAAGCCCCAAGAAATACAAGCACACCTGATAAGCGAGATTTAAGGATAACAGCGAAATTCAATAGGGTCTGAATTTCCAAACTAGGTTAAATGCCACGACGTTTTATTGTTGCCCCATTCAAGCAACATTTGAGAACCGAATAGAAATCTTTTAGTAAAAAGCGTTCTTTTTTGGGTCAGCGGTTAATGTGGACGGTTTAACGGTTTTTCCCCTGCGGGTCGTATTGGAAAGCCATTGAAAAGCTGATGGATAACTCTGCGAGTTACCCACGAGCTTTCCAACAGCTTTCCAACACTAAAAACCTACCGCCCACAATAACCACTTCCCTAATAATAAAATTTTTTTATTTTTATTTTGGTTCAAAGGCTCACGATGTTCGCCTAATAAAACGAAGTCGCCTATCGGCTCCGCTGATTTTTATATATCACTCTCGGGGCTTTTGGTGTACTATTGTCTTTTGTAATAGCAAGGACACAAAAAGGGTACTCTTCGAGTTTCCTTTTTGACCTTGCAAAAGGGCTTTGCCCCCTTGACCCCCGACCGCTTTCAGCGGTCAAAATAGAAGAACGGACACCATTATGAAACGTGAGAAAGAGATAAAAATCAGGCTCACCGAAAACGAGTATCAAGCCTTGTTAGAGAGAAAAACGAAAGCAAGGCTTGCGGAGTGGGTTCGGGAAGTTGCCCTGGAACAGCAACCTAAGCGACAGCCGAAAGTAATCGACCCTGCGTTACTGTTCGAGCTGAACCGCATAGGCGTAAACCTGAACCAAATCGCCCGACAATGCAACAGTCAAAAGCCGAGCATTGACCTTGTTAGCGTGTTGGCGACCTTGCGAGAAATTGAAAAAAATCTCAAAAAATTGCGAGAATTGAGCCTATGATCGAATTCaaacgtgattttttaaggcgttagtattattattaaggtattttttcaattcttatcagatttatttactcatagaggataggtatgcaacaccttaaagacattacagaacaggcaaaagacgcgattgagaaattgcacgataaaagcctcgaaaccctagatgcgattcgcgtagaatattttggtaaaaaagggcattttacccaattaatgcaagggttgcgtgatattgccgccgaagagcgtcctgcaatgggggcgaaaattaatgaggcaaaacagaaagttttagacttattaaacagcaaaaaagcggaatgggagcaagaagccttagatgctcagcttgccaaagaaagtattgacgtgagcttgccagggcgtaaaacggaacttggtggcttgcaccctgtttctgtaactattgggcgtgtggtgagctttttctctaatttaggttttacggtggaagtggggcctgaaattgaaacggattattacaattttgatgcgttaaatatccccgctcatcacccagcccgtgccgatcacgataccttctggtttgatgcacagcgtttattgcgtacccaaacctctggggtgcaaattcgtacaatggaaaaagccaaaccacctatccgtattatcgcccctgggcgtgtttatcgtaatgattacgaccaaacccacaccccaatgttccaccaaattgaattgctttatgtggataagcacgccaatttcaccgaattaaaaggcttgttacacgatttcttgcgtgcattctttgaagaagatttaaaagtgcgtttccgcccgtcttatttcccattcactgagccttctgctgaggttgatgtaatggggaaaaatggcaaatggcttgaagtgttaggttgtggaatggttcacccaaatgtgttacgcaatgtggggattgatccagaagaatattctggctttggggtgggtatgggcgtagagcgtttaacaatgttacgttacaatgtaacagatttgcgttcattctttgaaaacgatctgcgtttcttaaaacaatttaaataaCTCGAGttaatccgattgaccgaataaaatttacgatagagacgatCGGTGCGGGCCTCTTCGCTATTACGCCAGCTGGCGAAAGGGGGATGTGCTGCAAGGCGATTAAGTTGGGTAACGCCAGGGTTTTCCCAGTCACGACGTTGTAAAACGACGGCCAGTGAATTTAGGTGACACTATAGAATACTCAAGCTTGCATGCCTGCAGGTCGACTCTAGAGGATCCCCGGGTACCGAGCTCGAATTGACATAAGCCTGTTCGGTTCGTAAACTGTAATGCAAGTAGCGTATGCGCTCACGCAACTGGTCCAGAACCTTGACCGAACGCAGCGGTGGTAACGGCGCAGTGGCGGTTTTCATGGCTTGTTATGACTGTTTTTTTGTACAGTCTATGCCTCGGGCATCCAAGCAGCAAGCGCGTTACGCCGTGGGTCGATGTTTGATGTTATGGAGCAGCAACGATGTTACGCAGCAGCAACGATGTTACGCAGCAGGGCAGTCGCCCTAAAACAAAGTTAGGTGGCTCAAGTATGGGCATCATTCGCACATGTAGGCTCGGCCCTGACCAAGTCAAATCCATGCGGGCTGCTCTTGATCTTTTCGGTCGTGAGTTCGGAGACGTAGCCACCTACTCCCAACATCAGCCGGACTCCGATTACCTCGGGAACTTGCTCCGTAGTAAGACATTCATCGCGCTTGCTGCCTTCGACCAAGAAGCGGTTGTTGGCGCTCTCGCGGCTTACGTTCTGCCCAGGTTTGAGCAGCCGCGTAGTGAGATCTATATCTATGATCTCGCAGTCTCCGGCGAGCACCGGAGGCAGGGCATTGCCACCGCGCTCATCAATCTCCTCAAGCATGAGGCCAACGCGCTTGGTGCTTATGTGATCTACGTGCAAGCAGATTACGGTGACGATCCCGCAGTGGCTCTCTATACAAAGTTGGGCATACGGGAAGAAGTGATGCACTTTGATATCGACCCAAGTACCGCCACCTAACAATTCGTTCAAGCCGAGATCGGCTTCCCGGCCGCGGAGTTGTTCGGTAAATTGTCACAACGCCGCGGCCAATTCGAGCTCGGTACCCGGGGATCCTCTAGAGTCGACCTGCAGGCATGCAAGCTTGTCTCCCTATAGTGAGTCGTATTAGAGCTTGGCGTAATCATGGTCATAGCTGTTTCCTGTGTGAAATTGTTATCCGCTCACAATTCCACACAACATACGAGCCGGAAGCATAAAGTGTAAAGCCTGGGGTGCCTAATGAGTGAGCTAACTCACATTAATTGCGTTGCGCTCACTGCCCGCTTTCGAGTCGGGAAACCTGTCGTGCCAGCTGCATTAATGAATCGGCCAACGCGCGGGGAGAGGCGGTTTGCGTATTGGGCGCTCTTCCGCTTCCTCGCTCACTGACTCGCTGCGCTCGGTCGTTCGGCTGCGGCGAGCGGTATCAGCTCACTCAAAGGCGGTAATACGGTTATCCACAGAATCAGGGGATAACGCAGGAAAGAACATGTGAGCAAAAGGCCAGCAAAAGGCCAGGAACCGTAAAAAGGCCGCGTTGCTGGCGTTTTTCGATAGGCTCCGCCCCCCTGACGAGCATCACAAAAATCGACGCTCAAGTCAGAGGTGGCGAAACCCGACAGGACTATAAAGATACCAGGCGTTTCCCCCTGGAAGCTCCCTCGTGCGCTCTCCTGTTCCGACCCTGCCGCTTACCGGATACCTGTCCGCCTTTCTCCCTTCGGGAAGCGTGGCGCTTTCTCATAGCTCACGCTGTAGGTATCTCAGTTCGGTGTAGGTCGTTCGCTCCAAGCTGGGCTGTGTGCACGAACCCCCCGTTCAGCCCGACCGCTGCGCCTTATCCGGTAACTATCGTCTTGAGTCCAACCCGGTAAGACACGACTTATCGCCACTGGCAGCAGCCACTGGTAACAGGATTAGCAGAGCGAGGTATGTAGGCGGTGCTACAGAGTTCTTGAAGTGGTGGCCTAACTACGGCTACACTAGAAGGACAGTATTTGGTATCTGCGCTCTGCTGAAGCCAGTTACCTTCGGAAAAAGAGTTGGTAGCTCTTGATCCGGCAAACAAACCACCGCTGGTAGCGGTGGTTTTTTTGTTTGCAAGCAGCAGATTACGCGCAGAAAAAAAGGATCTCAAGAAGATCCTTTGATCTTTTCTACGGGGTCTGACGCTCAGTGGAACGAAAACTCACGTTAAGGGATTTTGGTCATGAGATTATCAAAAAGGATCTTCACCTAGATCCTTTTAAATTAAAAATGAAGTTTTAAATCAATCTAAAGT |
| pJSF02 (*ApglgB* mut) | GACGAAAGGGCCTCGTGATACGCCTATTTTTATAGGTTAATGTCATGATAATAATGGTTTCTTAGACGTCAGGTGGCACTTTTCGGGGAAATGTGCGCGGAACCCCTATTTGTTTATTTTTCTAAATACATTCAAATATGTATCCGCTCATGAGACAATAACCCTGATAAATGCTTCAATAATATTGAAAAAGGAAGAGTATGAGTATTCAACATTTCCGTGTCGCCCTTATTCCCTTTTTTGCGGCATTTTGCCTTCCTGTTTTTGCTCACCCAGAAACGCTGGTGAAAGTAAAAGATGCTGAAGATCAGTTGGGTGCACGAGTGGGTTACATCGAACTGGATCTCAACAGCGGTAAGATCCTTGAGAGTTTTCGCCCCGAAGAACGTTTTCCAATGATGAGCACTTTTAAAGTTCTGCTATGTGGCGCGGTATTATCCCGTATTGACGCCGGGCAAGAGCAACTCGGTCGCCGCATACACTATTCTCAGAATGACTTGGTTGAGTACTCACCAGTCACAGAAAAGCATCTTACGGATGGCATGACAGTAAGAGAATTATGCAGTGCTGCCATAACCATGAGTGATAACACTGCGGCCAACTTACTTCTGACAACGATCGGAGGACCGAAGGAGCTAACCGCTTTTTTGCACAACATGGGGGATCATGTAACTCGCCTTGATCGTTGGGAACCGGAGCTGAATGAAGCCATACCAAACGACGAGCGTGACACCACGATGCCTGTAGCAATGGCAACAACGTTGCGCAAACTATTAACTGGCGAACTACTTACTCTAGCTTCCCGGCAACAATTAATAGACTGGATGGAGGCGGATAAAGTTGCAGGACCACTTCTGCGCTCGGCCCTTCCGGCTGGCTGGTTTATTGCTGATAAATCTGGAGCCGGTGAGCGTGGGTCTCGCGGTATCATTGCAGCACTGGGGCCAGATGGTAAGCCCTCCCGTATCGTAGTTATCTACACGACGGGGAGTCAGGCAACTATGGATGAACGAAATAGACAGATCGCTGAGATAGGTGCCTCACTGATTAAGCATTGGTAACTGTCAGACCAAGTTTACTCATATATACTTTAGATTGATTTAAAACTTCATTTTTAATTTAAAAGGATCTAGGTGAAGATCCTTTTTGATAATCTCATGACCAAAATCCCTTAACGTGAGTTTTCGTTCCACTGAGCGTCAGACCCCGTAGAAAAGATCAAAGGATCTTCTTGAGATCCTTTTTTTCTGCGCGTAATCTGCTGCTTGCAAACAAAAAAACCACCGCTACCAGCGGTGGTTTGTTTGCCGGATCAAGAGCTACCAACTCTTTTTCCGAAGGTAACTGGCTTCAGCAGAGCGCAGATACCAAATACTGTTCTTCTAGTGTAGCCGTAGTTAGGCCACCACTTCAAGAACTCTGTAGCACCGCCTACATACCTCGCTCTGCTAATCCTGTTACCAGTGGCTGCTGCCAGTGGCGATAAGTCGTGTCTTACCGGGTTGGACTCAAGACGATAGTTACCGGATAAGGCGCAGCGGTCGGGCTGAACGGGGGGTTCGTGCACACAGCCCAGCTTGGAGCGAACGACCTACACCGAACTGAGATACCTACAGCGTGAGCTATGAGAAAGCGCCACGCTTCCCGAAGGGAGAAAGGCGGACAGGTATCCGGTAAGCGGCAGGGTCGGAACAGGAGAGCGCACGAGGGAGCTTCCAGGGGGAAACGCCTGGTATCTTTATAGTCCTGTCGGGTTTCGCCACCTCTGACTTGAGCGTCGATTTTTGTGATGCTCGTCAGGGGGGCGGAGCCTATGGAAAAACGCCAGCAACGCGGCCTTTTTACGGTTCCTGGCCTTTTGCTGGCCTTTTGCTCACATGTTCTTTCCTGCGTTATCCCCTGATTCTGTGGATAACCGTATTACCGCCTTTGAGTGAGCTGATACCGCTCGCCGCAGCCGAACGACCGAGCGCAGCGAGTCAGTGAGCGAGGAAGCGGAAGAGCGCCCAATACGCAAACCGCCTCTCCCCGCGCGTTGGCCGATTCATTAATGCAGCTGGCACGACAGGTTTCCCGACTGGAAAGCGGGCAGTGAGCGCAACGCAATTAATGTGAGTTAGCTCACTCATTAGGCACCCCAGGCTTTACACTTTATGCTTCCGGCTCGTATGTTGTGTGGAATTGTGAGCGGATAACAATTTCACACAGGAAACAGCTATGACCATGATTACGCCAAGCTTGCATGCCTGCAGGTCGACTCTAGAGGtgcaccgcactttGTATTATTGCCTGATGCCAATCAGGTTAAGGTGCTGGATAAAGAAAATCCAAGTAAGGTTTATCCCTTGGATTGCCTTGATGAACGTGGCTTTTTTGCGGGTATCATTCCAAACACACACAGTTTTTTTGCTTATCAATTAGAAGTCTATTGGGGCAATGAACCACAGATTGTAGAAGATCCGTATCGTTTTCACCCAATGATTCAAGAGCTGGATAATTGGCTATTGGCGGAGGGTTCTCATTTACGCCCTTATGAAATTTTAGGCGCACACTTTATGCAATGCGAAGGCGTATCAGGCGTGAATTTCCGCTTATGGGCGCCGAATGCGAAGCGGGTTTCGGTGGTGGGGGATTTCAACTATTGGGACGGTCGCCGCCACCCGATGCGTTTTCATTCCTCAAGTGGCATTTGGGAGCTGTTTATCCCTAAAGTGGCACTGGGGCAGTTGTATAAATTTGAATTATTGGATTGTAACGATCAGCTCCGTTTGAAAGCCGACCCTTATGCTTTTAGCTCACAGCTTCGTCCTGATACGGCTTCGCAAATTAGCGTATTGCCGAATGTAGTGGAAATGACGGAAAAACGTCGCAAAGCCAACCAGTTTGATCAGCCGATTTCCATTTATGAAGTGCATTTAGGCTCTTGGCGGCGAAATCTCGCGAACAATTTTTGGTTAGATTACGATGAAATCGCTGATGAACTGATCCCTTATGTGAAAGAAATGGGCTTCACCCATATTGAATTTTTGCCGTTGTCAGAATTTCCATTTGACGGCTCTTGGGGCTATCAGCCAATTGGACTTTATTCGCCAACCAGCCGTTTTGGCACACCTGAAGGCTTTAAACGCTTAGTCGATAAAGCTCACGAAGCAGGCATTAATGTGATTTTAGATTGGGTACCAGGGCATTTCCCAAGTGATACCGACGTCCTAGCATCACTGAGAAACGTGATTTTTTAAGGCGTTAGTATTATTATTAAGGTATTTTTTCAATTCTTATCAGATTTATTTACTCATAGAGGATAGGTatgcaacaccttaaagacattacagaacaggcaaaagacgcgattgagaaattgcacgataaaagcctcgaaaccctagatgcgattcgcgtagaatattttggtaaaaaagggcattttacccaattaatgcaagggttgcgtgatattgccgccgaagagcgtcctgcaatgggggcgaaaattaatgaggcaaaacagaaagttttagacttattaaacagcaaaaaagcggaatgggagcaagaagccttagatgctcagcttgccaaagaaagtattgacgtgagcttgccagggcgtaaaacggaacttggtggcttgcaccctgtttctgtaactattgggcgtgtggtgagctttttctctaatttaggttttacggtggaagtggggcctgaaattgaaacggattattacaattttgatgcgttaaatatccccgctcatcacccagcccgtgccgatcacgataccttctggtttgatgcacagcgtttattgcgtacccaaacctctggggtgcaaattcgtacaatggaaaaagccaaaccacctatccgtattatcgcccctgggcgtgtttatcgtaatgattacgaccaaacccacaccccaatgttccaccaaattgaattgctttatgtggataagcacgccaatttcaccgaattaaaaggcttgttacacgatttcttgcgtgcattctttgaagaagatttaaaagtgcgtttccgcccgtcttatttcccattcactgagccttctgctgaggttgatgtaatggggaaaaatggcaaatggcttgaagtgttaggttgtggaatggttcacccaaatgtgttacgcaatgtggggattgatccagaagaatattctggctttggggtgggtatgggcgtagagcgtttaacaatgttacgttacaatgtaacagatttgcgttcattctttgaaaacgatctgcgtttcttaaaacaatttaaataaAGACTGAGCGTCGTGAGTACATGAACGAGAAAAATATAAAACACAGTCAAAACTTTATTACTTCAAAACATAATATAGATAAAATAATGACAAATATAAGATTAAATGAACATGATAATATCTTTGAAATCGGCTCAGGAAAAGGCCATTTTACCCTTGAATTAGTAAAGAGGTGTAATTTCGTAACTGCCATTGAAATAGACCATAAATTATGCAAAACTACAGAAAATAAACTTGTTGATCACGATAATTTCCAAGTTTTAAACAAGGATATATTGCAGTTTAAATTTCCTAAAAACCAATCCTATAAAATATATGGTAATATACCTTATAACATAAGTACGGATATAATACGCAAAATTGTTTTTGATAGTATAGCTAATGAGATTTATTTAATCGTGGAATACGGGTTTGCTAAAAGATTATTAAATACAAAACGCTCATTGGCATTACTTTTAATGGCAGAAGTTGATATTTCTATATTAAGTATGGTTCCAAGAGAATATTTTCATCCTAAACCTAAAGTGAATAGCTCACTTATCAGATTAAGTAGAAAAAAATCAAGAATATCACACAAAGATAAACAAAAGTATAATTATTTCGTTATGAAATGGGTTAACAAAGAATACAAGAAAATATTTACAAAAAATCAATTTAACAATTCCTTAAAACATGCAGGAATTGACGATTTAAACAATATTAGCTTTGAACAATTCTTATCTCTTTTCAATAGCTATAAATTATTTAATAAGTAACCAAATCGGATCCCGGGCCCGTCGACTGCAGAGGCCTGCATGCAAGCTTGGCGTAATCATGGTCATAGCTGTTTCCTGTGTGCTGAACTTCGTCACTCATCGGGCTAGAACGCTTTGGTTTAGATGGCTTGCGTGTGGACGCGGTGGCATCAATGATTTACCGTGATTACAGCCGTGCAGACGGCGAGTGGATTCCAAATCAATATGGCGGTCGGGAAAATTTAGAGGCGATCGAATTTTTAAAACACACCAATTATGTATTGGGAACAGAAAATGCGGGGGCGATCACCATTGCGGAAGAATCCACTTCTTTTGCGGGCGTTACGCACCCACCGCAAGATGGCGGGCTAGGTTTCCACTTCAAATGGAATATGGGGTGGATGAACGACACGCTATCCTATATGCAAAAAGATCCCATTTATCGCCAACATCACCACAGCCAAATGACCTTTGGAATGATGTATCAATACAGCGAAAATTTCGTGCTTCCTCTTTCGCACGATGAAGTGGTACACGGTAAAGGCTCACTTATCGGAAAAATGCCCGGTGATGCGTGGCAAAAATTTGCCAACCTACGGGCGTATTATGGCTATATGTGGGGCTATCCAGGGAAAAAATTGCTCTTTATGGGCAATGAATTTGCTCAGGGGCGAGAATGGAACTATCAAGAAAGCCTTGATTGGTTCTTGCTCGATGAAAATATCGGCGGCGGCTGGCATAACGGCGTACAAAAATTTGTGCAAGATTTGAACCGCACTTATCAAACGCACCCCGCTTTATATGAGTTGGATTATGACCCTGCAGGCTTTAATTGGCTGGTGGTGGACGACTATCAAAACTCCGTTTTTGTGTTTGAACGTTGTAGTCGTGATGGCGAACGCCTTATTGTGGTAAGCAACTTCACCCCCGTGCCTCGCTATAATTATCGTTTTGGCGTGAACGAAGCAGGGGAGTATGAAGAAATTCTTAATTCTGATTCGGCATTTTATCAAGGCTCAAATTTAGGTAATCAAGGCGTGGTGGTGAGTGAAGCCATCGGGGTACCGAGCTCGAATTCACTGGCCGTCGTTTTACAACGTCGTGACTGGGAAAACCCTGGCGTTACCCAACTTAATCGCCTTGCAGCACATCCCCCTTTCGCCAGCTGGCGTAATAGCGAAGAGGCCCGCACCGATCGCCCTTCCCAACAGTTGCGCAGCCTGAATGGCGAATGGCGCCTGATGCGGTATTTTCTCCTTACGCATCTGTGCGGTATTTCACACCGCATATGGTGCACTCTCAGTACAATCTGCTCTGATGCCGCATAGTTAAGCCAGCCCCGACACCCGCCAACACCCGCTGACGCGCCCTGACGGGCTTGTCTGCTCCCGGCATCCGCTTACAGACAAGCTGTGACCGTCTCCGGGAGCTGCATGTGTCAGAGGTTTTCACCGTCATCACCGAAACGCGCGA |
| pJSF05 (*FtlpxE*) | GACGAAAGGGCCTCGTGATACGCCTATTTTTATAGGTTAATGTCATGATAATAATGGTTTCTTAGACGTCAGGTGGCACTTTTCGGGGAAATGTGCGCGGAACCCCTATTTGTTTATTTTTCTAAATACATTCAAATATGTATCCGCTCATGAGACAATAACCCTGATAAATGCTTCAATAATATTGAAAAAGGAAGAGTATGAGTATTCAACATTTCCGTGTCGCCCTTATTCCCTTTTTTGCGGCATTTTGCCTTCCTGTTTTTGCTCACCCAGAAACGCTGGTGAAAGTAAAAGATGCTGAAGATCAGTTGGGTGCACGAGTGGGTTACATCGAACTGGATCTCAACAGCGGTAAGATCCTTGAGAGTTTTCGCCCCGAAGAACGTTTTCCAATGATGAGCACTTTTAAAGTTCTGCTATGTGGCGCGGTATTATCCCGTATTGACGCCGGGCAAGAGCAACTCGGTCGCCGCATACACTATTCTCAGAATGACTTGGTTGAGTACTCACCAGTCACAGAAAAGCATCTTACGGATGGCATGACAGTAAGAGAATTATGCAGTGCTGCCATAACCATGAGTGATAACACTGCGGCCAACTTACTTCTGACAACGATCGGAGGACCGAAGGAGCTAACCGCTTTTTTGCACAACATGGGGGATCATGTAACTCGCCTTGATCGTTGGGAACCGGAGCTGAATGAAGCCATACCAAACGACGAGCGTGACACCACGATGCCTGTAGCAATGGCAACAACGTTGCGCAAACTATTAACTGGCGAACTACTTACTCTAGCTTCCCGGCAACAATTAATAGACTGGATGGAGGCGGATAAAGTTGCAGGACCACTTCTGCGCTCGGCCCTTCCGGCTGGCTGGTTTATTGCTGATAAATCTGGAGCCGGTGAGCGTGGGTCTCGCGGTATCATTGCAGCACTGGGGCCAGATGGTAAGCCCTCCCGTATCGTAGTTATCTACACGACGGGGAGTCAGGCAACTATGGATGAACGAAATAGACAGATCGCTGAGATAGGTGCCTCACTGATTAAGCATTGGTAACTGTCAGACCAAGTTTACTCATATATACTTTAGATTGATTTAAAACTTCATTTTTAATTTAAAAGGATCTAGGTGAAGATCCTTTTTGATAATCTCATGACCAAAATCCCTTAACGTGAGTTTTCGTTCCACTGAGCGTCAGACCCCGTAGAAAAGATCAAAGGATCTTCTTGAGATCCTTTTTTTCTGCGCGTAATCTGCTGCTTGCAAACAAAAAAACCACCGCTACCAGCGGTGGTTTGTTTGCCGGATCAAGAGCTACCAACTCTTTTTCCGAAGGTAACTGGCTTCAGCAGAGCGCAGATACCAAATACTGTTCTTCTAGTGTAGCCGTAGTTAGGCCACCACTTCAAGAACTCTGTAGCACCGCCTACATACCTCGCTCTGCTAATCCTGTTACCAGTGGCTGCTGCCAGTGGCGATAAGTCGTGTCTTACCGGGTTGGACTCAAGACGATAGTTACCGGATAAGGCGCAGCGGTCGGGCTGAACGGGGGGTTCGTGCACACAGCCCAGCTTGGAGCGAACGACCTACACCGAACTGAGATACCTACAGCGTGAGCTATGAGAAAGCGCCACGCTTCCCGAAGGGAGAAAGGCGGACAGGTATCCGGTAAGCGGCAGGGTCGGAACAGGAGAGCGCACGAGGGAGCTTCCAGGGGGAAACGCCTGGTATCTTTATAGTCCTGTCGGGTTTCGCCACCTCTGACTTGAGCGTCGATTTTTGTGATGCTCGTCAGGGGGGCGGAGCCTATGGAAAAACGCCAGCAACGCGGCCTTTTTACGGTTCCTGGCCTTTTGCTGGCCTTTTGCTCACATGTTCTTTCCTGCGTTATCCCCTGATTCTGTGGATAACCGTATTACCGCCTTTGAGTGAGCTGATACCGCTCGCCGCAGCCGAACGACCGAGCGCAGCGAGTCAGTGAGCGAGGAAGCGGAAGAGCGCCCAATACGCAAACCGCCTCTCCCCGCGCGTTGGCCGATTCATTAATGCAGCTGGCACGACAGGTTTCCCGACTGGAAAGCGGGCAGTGAGCGCAACGCAATTAATGTGAGTTAGCTCACTCATTAGGCACCCCAGGCTTTACACTTTATGCTTCCGGCTCGTATGTTGTGTGGAATTGTGAGCGGATAACAATTTCACACAGGAAACAGCTATGACCATGATTACGCCAAGCTTGCATGCCTGCAGGTCGACTCTAGAGGTGCACCGCACTTTGTATTATTGCCTGATGCCAATCAGGTTAAGGTGCTGGATAAAGAAAATCCAAGTAAGGTTTATCCCTTGGATTGCCTTGATGAACGTGGCTTTTTTGCGGGTATCATTCCAAACACACACAGTTTTTTTGCTTATCAATTAGAAGTCTATTGGGGCAATGAACCACAGATTGTAGAAGATCCGTATCGTTTTCACCCAATGATTCAAGAGCTGGATAATTGGCTATTGGCGGAGGGTTCTCATTTACGCCCTTATGAAATTTTAGGCGCACACTTTATGCAATGCGAAGGCGTATCAGGCGTGAATTTCCGCTTATGGGCGCCGAATGCGAAGCGGGTTTCGGTGGTGGGGGATTTCAACTATTGGGACGGTCGCCGCCACCCGATGCGTTTTCATTCCTCAAGTGGCATTTGGGAGCTGTTTATCCCTAAAGTGGCACTGGGGCAGTTGTATAAATTTGAATTATTGGATTGTAACGATCAGCTCCGTTTGAAAGCCGACCCTTATGCTTTTAGCTCACAGCTTCGTCCTGATACGGCTTCGCAAATTAGCGTATTGCCGAATGTAGTGGAAATGACGGAAAAACGTCGCAAAGCCAACCAGTTTGATCAGCCGATTTCCATTTATGAAGTGCATTTAGGCTCTTGGCGGCGAAATCTCGCGAACAATTTTTGGTTAGATTACGATGAAATCGCTGATGAACTGATCCCTTATGTGAAAGAAATGGGCTTCACCCATATTGAATTTTTGCCGTTGTCAGAATTTCCATTTGACGGCTCTTGGGGCTATCAGCCAATTGGACTTTATTCGCCAACCAGCCGTTTTGGCACACCTGAAGGCTTTAAACGCTTAGTCGATAAAGCTCACGAAGCAGGCATTAATGTGATTTTAGATTGGGTACCAGGGCATTTCCCAAGTGATACCGACGTCCTAGCATCACTGAGTAGATTAGAGTAATCAAGTCAATAGTTTCTAATATTTGTTTAACAATCTTTGATCGATTGGCTATAGGCTAGTGAAATAATAATATTTTGTTATACTAAGCTTGTAACTATCTAATTAATAGGAAAATTTTATATatgctcaaacagacattacaaacaaactttcaaggttttaaagatatttttaaaaaaccaaaactacacaatcataaattgcctagatatctacagttgaaatatacgtttataccattattaattttggtaatttttgcatactataacttagataccccagttgagaactatatcaagcattctatgccaaatattgttggtgtaatttttggtaaaataactgatgttggtaaggccgagtatattttgataatttgcggtgtgatagtgttagcgcgtttatttacagatagccaaaaattatctgctaatactagagctatgtttgacaaggtgtcggcatatgcgggttttatcttagcaactgtagctattagtggtattttgggacaaatactcaagatgataataggtagagcgcgtcctaagtttttcttggaatatggttcgcattatttccaacattttcatgcacctggatatgattttgcaagtatgccgtcagggcactcaatcacagttggagcaatgtttatagcatttttttatattttccctaagctaagatatttttggtatttgctgatagtggtatttgctgggagtagaattatggttggttcacattatcctagtgatgtaatttttggcgttgcttttggttgttactgtacagcatatatctactattggatgagaaatagagagattatttagCTGAACTTCGTCACTCATCGGGCTAGAACGCTTTGGTTTAGATGGCTTGCGTGTGGACGCGGTGGCATCAATGATTTACCGTGATTACAGCCGTGCAGACGGCGAGTGGATTCCAAATCAATATGGCGGTCGGGAAAATTTAGAGGCGATCGAATTTTTAAAACACACCAATTATGTATTGGGAACAGAAAATGCGGGGGCGATCACCATTGCGGAAGAATCCACTTCTTTTGCGGGCGTTACGCACCCACCGCAAGATGGCGGGCTAGGTTTCCACTTCAAATGGAATATGGGGTGGATGAACGACACGCTATCCTATATGCAAAAAGATCCCATTTATCGCCAACATCACCACAGCCAAATGACCTTTGGAATGATGTATCAATACAGCGAAAATTTCGTGCTTCCTCTTTCGCACGATGAAGTGGTACACGGTAAAGGCTCACTTATCGGAAAAATGCCCGGTGATGCGTGGCAAAAATTTGCCAACCTACGGGCGTATTATGGCTATATGTGGGGCTATCCAGGGAAAAAATTGCTCTTTATGGGCAATGAATTTGCTCAGGGGCGAGAATGGAACTATCAAGAAAGCCTTGATTGGTTCTTGCTCGATGAAAATATCGGCGGCGGCTGGCATAACGGCGTACAAAAATTTGTGCAAGATTTGAACCGCACTTATCAAACGCACCCCGCTTTATATGAGTTGGATTATGACCCTGCAGGCTTTAATTGGCTGGTGGTGGACGACTATCAAAACTCCGTTTTTGTGTTTGAACGTTGTAGTCGTGATGGCGAACGCCTTATTGTGGTAAGCAACTTCACCCCCGTGCCTCGCTATAATTATCGTTTTGGCGTGAACGAAGCAGGGGAGTATGAAGAAATTCTTAATTCTGATTCGGCATTTTATCAAGGCTCAAATTTAGGTAATCAAGGCGTGGTGGTGAGTGAAGCCATCGGGGTACCGAGCTCGAATTCACTGGCCGTCGTTTTACAACGTCGTGACTGGGAAAACCCTGGCGTTACCCAACTTAATCGCCTTGCAGCACATCCCCCTTTCGCCAGCTGGCGTAATAGCGAAGAGGCCCGCACCGATCGCCCTTCCCAACAGTTGCGCAGCCTGAATGGCGAATGGCGCCTGATGCGGTATTTTCTCCTTACGCATCTGTGCGGTATTTCACACCGCATATGGTGCACTCTCAGTACAATCTGCTCTGATGCCGCATAGTTAAGCCAGCCCCGACACCCGCCAACACCCGCTGACGCGCCCTGACGGGCTTGTCTGCTCCCGGCATCCGCTTACAGACAAGCTGTGACCGTCTCCGGGAGCTGCATGTGTCAGAGGTTTTCACCGTCATCACCGAAACGCGCGA |
|  |  |

**Figure S1**


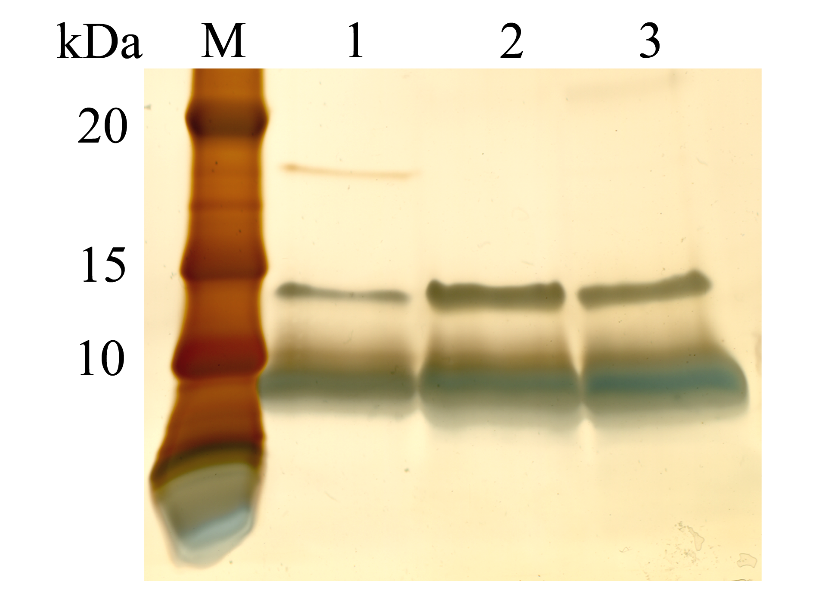

Supplement: Supplemental material — Tables S1 and S2; Fig. S1. [file aem.02398-25-s0002.docx]
